# Intimomedial tears of the aorta heal by smooth muscle cell–mediated fibrosis without atherosclerosis

**DOI:** 10.1172/jci.insight.172437

**Published:** 2024-04-09

**Authors:** Abdulrahman H.M. Hassab, David J. Hur, Prashanth Vallabhajosyula, George Tellides, Roland Assi

**Affiliations:** 1 Department of Surgery (Cardiac), and; 2 Department of Internal Medicine (Cardiovascular Medicine), Yale School of Medicine, New Haven, Connecticut, USA.; 3 Veterans Affairs Connecticut Healthcare System, West Haven, Connecticut, USA.; 4 Program in Vascular Biology and Therapeutics, Yale School of Medicine, New Haven, Connecticut, USA.

**Keywords:** Vascular biology, Atherosclerosis

## Abstract

**BACKGROUND:**

Disease of the aorta varies from atherosclerosis to aneurysms, with complications including rupture, dissection, and poorly characterized limited tears. We studied limited tears without any mural hematoma, termed intimomedial tears, to gain insight into aortic vulnerability to excessive wall stresses. Our premise is that minimal injuries in aortas with sufficient medial resilience to prevent tear progression correspond to initial mechanisms leading to complete structural failure in aortas with significantly compromised medial resilience.

**METHODS:**

Intimomedial tears were macroscopically identified in 9 of 108 ascending aortas after surgery and analyzed by histology and immunofluorescence confocal microscopy.

**RESULTS:**

Nonhemorrhagic, nonatheromatous tears correlated with advanced aneurysmal disease and most lacked distinctive symptoms or radiological signs. Tears traversed the intima and part of the subjacent media, while the resultant defects were partially or completely filled with neointima characterized by differentiated smooth muscle cells, scattered leukocytes, dense fibrosis, and absent elastic laminae despite tropoelastin synthesis. Healed lesions contained organized fibrin at tear edges without evidence of plasma and erythrocyte extravasation or lipid accumulation.

**CONCLUSION:**

These findings suggest a multiphasic model of aortic wall failure in which primary lesions of intimomedial tears either heal if the media is sufficiently resilient or progress as dissection or rupture by medial delamination and tear completion, respectively. Moreover, mural incorporation of thrombus and cellular responses to injury, two historically important concepts in atheroma pathogenesis, contribute to vessel wall repair with adequate conduit function, but even together are not sufficient to induce atherosclerosis.

**FUNDING:**

NIH (R01-HL146723, R01-HL168473) and Yale Department of Surgery.

## Abstract 2

Smooth muscle cell responses to mechanical injury have a reparative role to prevent the progression of localized vessel wall failure as rupture or dissection.

## Graphical abstract

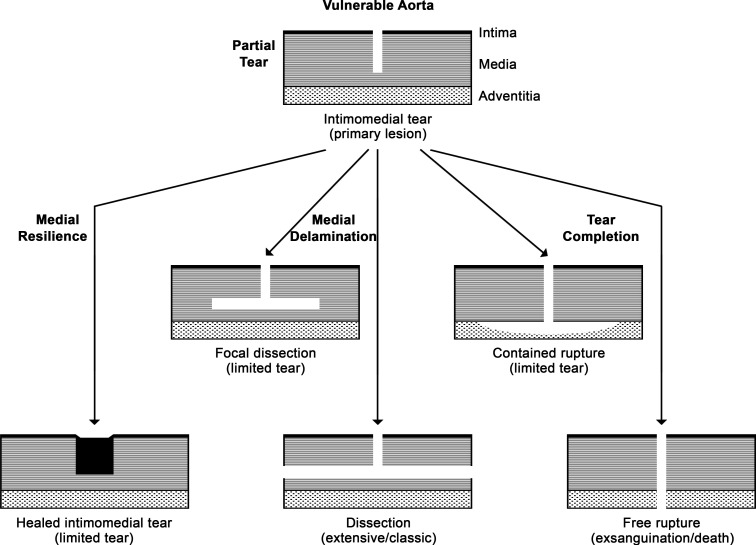

## Introduction

The aorta serves as a conduit to distribute oxygenated blood from the heart to organs and tissues of the body via arterial branches. Its vital function may be impaired by abnormalities of the vessel wall such as intimal plaques and medial degeneration leading to changes in aortic caliber of stenosis or aneurysm. Complications of bleeding or malperfusion can occur if aortic disease results in structural failure of the vessel wall, and the sudden presentation of these life-threatening problems are recognized as acute aortic syndrome (1). Structural failure of the aortic wall is classified as (i) rupture (transmedial or transmural tear), (ii) dissection (separation of the media forming a blood-filled channel that communicates with the lumen via an entry tear), (iii) intramural hematoma (separation of medial laminae filled with blood but without overt luminal communication), (iv) penetrating atherosclerotic ulcer (atheromatous plaque invading the media), and (v) poorly characterized lesions variously described as “limited intimal tear,” “limited dissection,” or “incomplete dissection” (tear through the intima and subjacent media without significant medial hematoma) (2, 3). Consensus nomenclature for the latter disorder includes “discrete/subtle dissection without haematoma,” “intimal tear without medial hematoma,” and “subtle or discrete aortic dissection with bulging of the aortic wall” (4–6). The terms “intimal” and “dissection” as descriptors are confusing, however, since the media is also involved and specifying without medial hematoma implies no dissection (though qualification “without significant medial hematoma” denotes that lesions with discrete medial hematoma or subadventitial hematoma not involving the media are included as limited tears). Thus, our preferred terminology for uncomplicated defects without progression to dissection or rupture and without any mural hematoma is “intimomedial tear” and we consider the less specific descriptor of “limited tear” to include intimomedial tear, focal dissection (circumscribed medial delamination of less than a few centimeters), and contained rupture (transmedial tear with bleeding restricted by adventitia and fibrotic perivascular tissue).

The concept of limited tears of the aorta was established over 50 years ago. Twelve cases were initially described in postmortem studies by the pathologist, Jesse Edwards (2). The lesions were distinguished from rupture and dissection as neither through-and-through tears nor with significant extravasation of blood into the media and were considered relatively benign conditions. Frequent association with aortic valve insufficiency was due to prolapse of the torn aortic wall and nearby valve leaflets. Temporal evolution of lesions was recognized, with tears of days in duration having distinct edges with little retraction versus adherent edges and with considerable retraction after weeks or longer. A further 9 cases were described in operative specimens by the surgeon, Lars Svensson (3). Clinical presentation varied from acute with chest pain and bloody pericardial effusion to chronic with no or mild symptoms and partially or fully healed tears. The lesions were missed by preoperative diagnostic imaging studies of computed tomography (CT) scans and transesophageal echocardiography (TEE). Technological advancements and/or broadening of definitions led to increased preoperative recognition of limited tears. Lesions presenting with chest pain and syncope were identified by TEE as bulging of the affected wall and discrete mural hematomas in 8 patients (7). Subadventitial blood and small dissection channels were found in the patients who underwent surgical repair, whereas progression to rupture or dissection occurred within hours in 2 patients managed medically. Additionally, limited tears presenting as acute aortic syndrome were diagnosed by CT angiography in 24 patients (8). The detected lesions were relatively large (1.7 to 7.5 cm) and most had associated findings, including intramural hematoma, periaortic fluid, and hemopericardium; a single healed tear, incidental to a separate acute lesion, was also described. Specimens from surgical repair showed focal dissection, while several patients treated medically survived.

Thus, the wide range in presentation, findings, and outcomes for limited tears raises the questions whether single or multiple conditions were considered and, if a distinct entity, how different pathological stages are related. Shortcomings to understanding the injury include overlooking subclinical lesions and a paucity of microscopic studies. To gain insight into mechanisms of aortic wall failure, we examined operative specimens for recent or healed intimomedial tears without any mural hematomas, i.e., not including focal dissection or contained rupture, and characterized cellular and extracellular matrix (ECM) changes by histology and immunofluorescence confocal microscopy.

## Results

### Nonhemorrhagic, nonatheromatous tears occur in a minority of diseased aortas.

**Figure 1:**
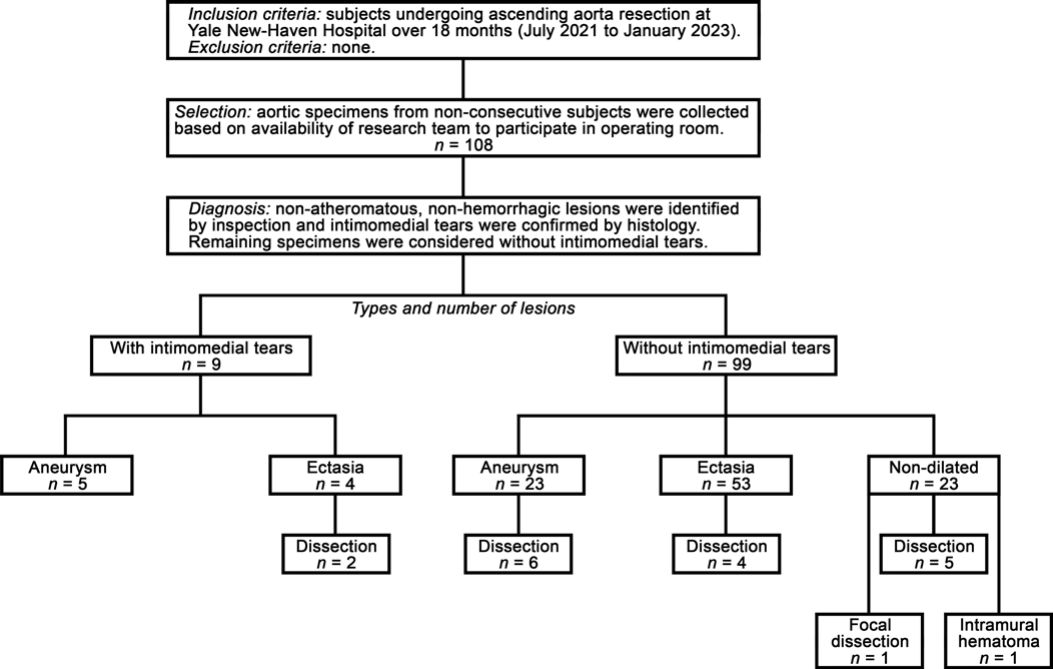
Flow diagram of observational study design, patients, and lesions. Summary of inclusion and exclusion criteria, selection of patients, diagnosis of lesions, and types and number of lesions. Ascending aortas were classified as aneurysm (>1.5× expected diameter), ectasia (>2 SD but ≤1.5× expected diameter), or nondilated (≤2 SD expected diameter) based on observed versus expected diameter normalized by patient age, sex, height, and weight. Dissection was diagnosed by clinical presentation, radiological imaging, and intraoperative mural hematoma, and were all classic extensive lesions, except for 1 focal dissection and 1 intramural hematoma. In addition to dissection, focal dissection, and intramural hematoma (*n* = 7), nondilated ascending aortas were associated with dilated aortic roots (*n* = 15) and posttraumatic aortic arch coarctation (*n* = 1). The latter case of a 29-year-old female without cardiovascular risk factors likely represents the only nondiseased ascending aorta in this series. No contained or free ruptures were encountered. Two intimomedial tears resulting in aortic valve insufficiency that were not excised but incorporated in the repair procedure were not part of the study, as aortic tissue was not excised.

**Figure 2:**
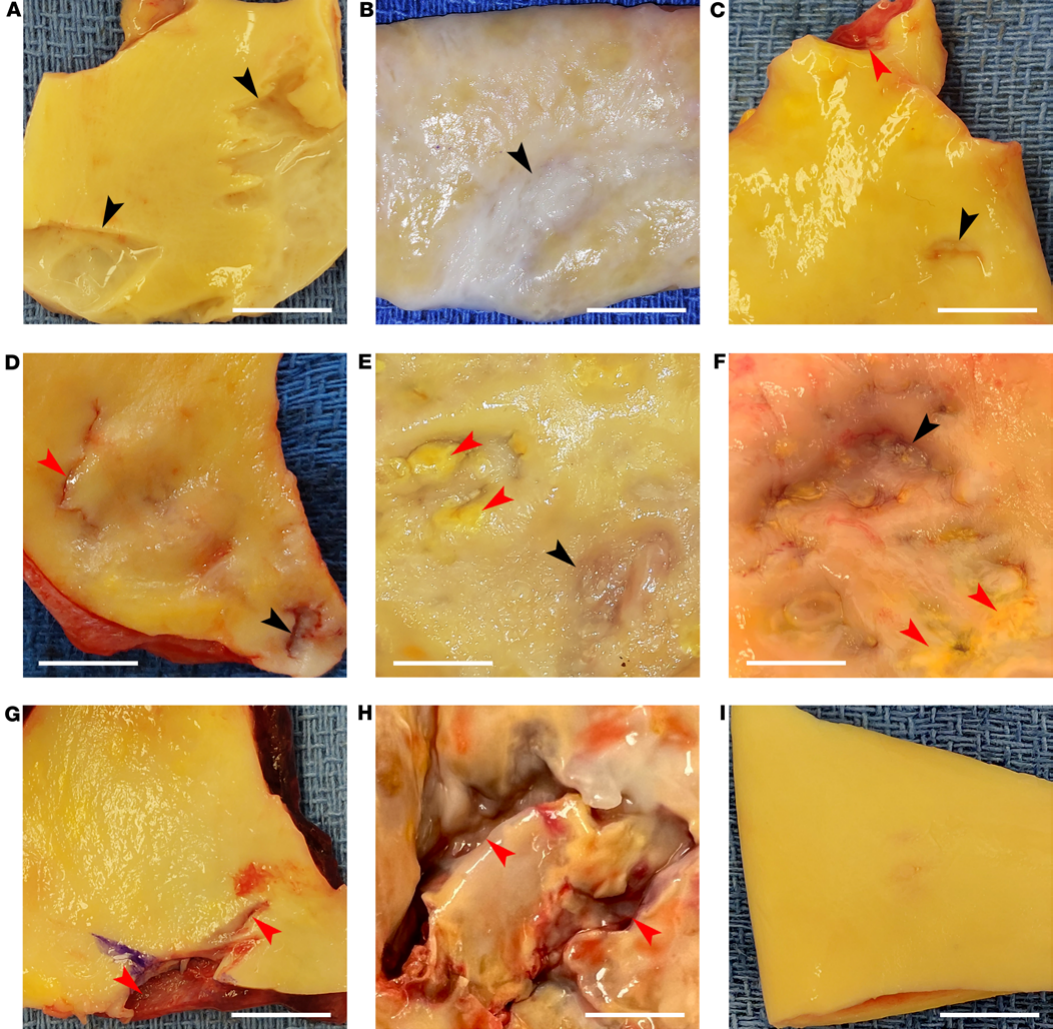
Macroscopic appearance of aortic tears. Operative specimens from patients undergoing surgery with ascending aorta resection were inspected for intimal defects. Nonhemorrhagic, nonatheromatous lesions had varying appearances of (**A**) multiple craters with corrugated or undermined edges, (**B**) white, scarred depression, (**C**) dimple with smooth rounded edges (lower arrow) not communicating with nearby dissection (upper arrow), (**D**) dimple with irregular edges (lower arrow) not communicating with nearby dissection and its entry tear (upper arrow), (**E**) pale depression (lower arrow) with nearby atheromatous plaques (upper arrows), and (**F**) pale depression (upper arrow) with nearby atheromatous plaques (lower arrows). Intimomedial tears were readily differentiated from (**G**) entry tear (upper arrow) communicating with dissection visible at cut edge of inner media (lower arrow), (**H**) penetrating atherosclerotic ulcer (arrows), and (**I**) smooth inner surface of aneurysms without intimal defects. The location of intimomedial tears varied from proximal (**A**, **B**, **E**, and **F**) to distal (**C**) ascending aorta and distal aortic root (**D**). Black arrows indicate intimomedial tears (isolated lesions in **A** and **B** near, but not communicating with, dissections in **C** and **D**, and near, but separate from, atherosclerotic plaques in **E** and **F**, while red arrows indicate lesions other than intimomedial tears. Orientation: proximal aorta below and distal aorta above. Scale bars: 1 cm.

To identify poorly characterized intimomedial tears, we inspected ascending aorta specimens, and the adjoining aortic root or aortic arch when included, from patients undergoing surgery with ascending aorta resection using an observational study design (Figure 1). Intimal defects, other than dissection entry tears, raised atheromatous plaques, and penetrating atherosclerotic ulcers, were found in 9 of 108 (8.3%) aortas (Supplemental Table 1; supplemental material available online with this article; https://doi.org/10.1172/jci.insight.172437DS1). Five (55.6%) were in the proximal ascending aorta, 1 (11.1%) in the mid ascending aorta, 1 (11.1%) in the distal ascending aorta, and 2 (22.2%) in the distal aortic root. The circumferential location varied from anterior to posterior surfaces and greater to lesser curvatures. Lesions were often multiple and ranged in size from 1 to 20 mm. Their shape varied from small dimples to large depressions to narrow crevices to broad craters. Lesion bases were usually a normal yellow-orange color, while some were discolored white and others appeared pale, almost translucent, when significantly thinned. The edges varied from smooth and rounded to corrugated and undermined. Four (44.4%) specimens contained isolated intimomedial tears, while 2 (22.2%) specimens had nearby dissections, and 3 (33.3%) specimens had nearby atheromatous plaques (Figure 2, A–F). Intimomedial tears were readily differentiated from dissection entry tears or penetrating atherosclerotic ulcers and differed from the smooth inner surface of aortic aneurysms without intimal defects (Figure 2, G–I). A variant type of limited tear of a focal dissection of the distal ascending aorta with 7.5 mm medial delamination and discrete medial hematoma was not included and no contained ruptures were encountered during the study period. In summary, intimomedial tears were found in a small number of diseased aortas in our series of patients.

### Intimomedial tears correlate with advanced aneurysmal disease and most lack distinctive symptoms or radiological signs.

Clinical characteristics were compared among patients with and without intimomedial tears of the proximal aorta (Table 1). Intimomedial tears associated with aortic dilatation (increased frequency of aneurysm diagnosis, increased aortic diameter, larger fold increase, and higher *z* score when aortic size was indexed to age, sex, and body mass), extensive aneurysmal disease (increased incidence of concomitant descending thoracic or abdominal aortic aneurysms), and aortic valve regurgitation (often secondary to aortic root dilatation but also associated with altered hemodynamic stresses). Other patient characteristics and features of arterial disease did not differ between the groups. In the group with intimomedial tears (Supplemental Table 1), chest pain was the presenting symptom in 2 (22.2%) patients, with concomitant aortic dissection and a recent symptom in 1 (11.1%) patient during a hypertensive crisis 3 months prior to surgery that resolved with antihypertensives and non-narcotic analgesics. Preoperative assessment by CT scan found subtle mural defects in 2 (22.2%) patients, vague or no abnormalities in 5 (55.6%) patients, and unable to evaluate the aortic wall because of insufficient quality in 2 (22.2%) patients (Supplemental Figure 1, A–C). On review, aneurysms and dissections were correctly diagnosed, but neither of the subtle contour abnormalities were described in routine clinical interpretations. Additionally, intraoperative assessment by TEE showed mild systolic wall bulging at lesion sites in 2 (22.2%) patients, but no specific abnormalities or did not visualize lesion sites in 7 (77.8%) patients (Supplemental Figure 1, D and E). In the patient with subacute chest pain and recent hypertensive crisis and in 1 asymptomatic patient, serial preoperative imaging revealed unusually rapid growth rates of approximately 1 cm/yr. Thus, intimomedial tears identified by gross inspection of surgical specimens were largely clinically inapparent, though some associated with accelerated aortic dilatation.

### Intimomedial tears traverse the intima and varying thickness of subjacent media, with incomplete healing distinguished by absent elastic laminae.

**Figure 3:**
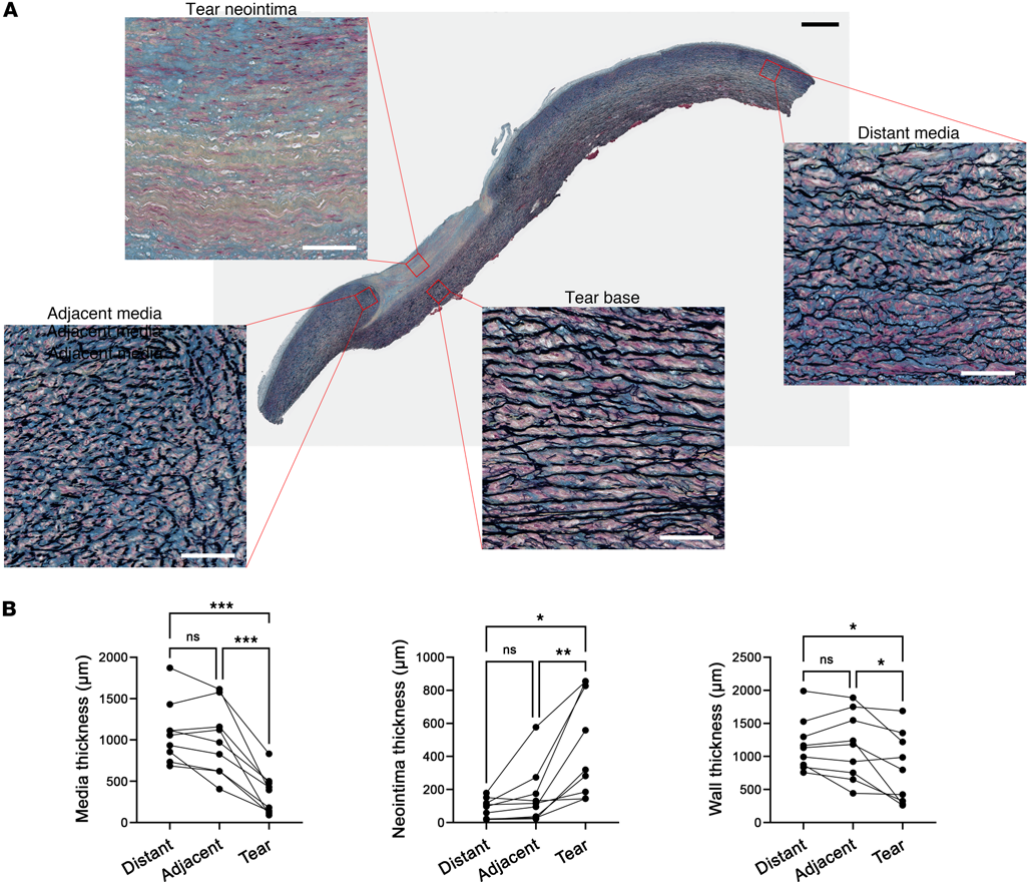
Vessel wall microstructure of intimomedial tears. Aortas with intimomedial tears were analyzed by histology using Movat’s pentachrome stain. (**A**) Typical chronic lesion with a wide tear separating the inner half of the media. The defect is filled by a thick neointima distinguished by increased collagen (yellow) and an absence of elastic laminae (black). Minimal neointima lines the luminal surface remote from the tear. The outer media at the base of the tear has intact elastic laminae similar to that of the adjacent and distant media. The left edge appears rolled up, with an underlying neointima attaching it to the tear base representing a healed focal dissection; the right edge has a clean break and is not undermined. Black scale bar: 1 mm; white scale bars: 100 μm. (**B**) Thickness measurements of media, neointima, and wall (media + neointima) at different sites of distant media, adjacent media, and intimomedial tear from single lesions of 9 patients. Data are means of 3 measurements, with lines connecting values from individual specimens. NS, not significant. *P < 0.05; **P < 0.01; ***P < 0.001 by repeated measures ANOVA with Tukey’s multiple-comparison test.

We analyzed vessel wall microstructure of intimomedial tears by histology. The media was torn, as evidenced by a focal absence of elastic laminae in the inner aortic wall (Figure 3A). Intact elastic laminae at either side of the lesion, termed “adjacent media,” suggested an initial radial tear of the aortic wall. The tears did not extend through the entire media, as intact elastic laminae were observed in the outer media, termed “tear base.” The lesion cavity was filled with cells and ECM not containing elastic laminae, termed “tear neointima.” The aortic wall remote from lesions, termed “distant media,” displayed fewer histological abnormalities. Most intimal tears exhibited a chronic healed appearance, with adherent edges and abundant neointima (Supplemental Figure 2A). Crater-like lesions in the patient with transient chest pain and previous hypertensive crisis were exceptions, with free (nonadherent) edges and thin neointima overlying the exposed media, suggesting subacute injury, but may reflect deficient repair (Supplemental Figure 2B). In contrast, acute intimomedial tears forming dissection entry sites had no neointima formation and minimal separation of the adjacent media (Supplemental Figure 2C). Histomorphometry of lesions confirmed that the tear base was thinner than the adjacent or distant media, that the tear neointima was markedly thicker than neointima overlying the adjacent and distant media, and that the combined thickness of the tear base and neointima was overall attenuated compared with the adjacent and distant aorta (Figure 3B). Extrapolation of the thickness of the tear base to that of the adjacent and distant media suggested that between 42% and 92% of the original media had torn. These data show that intimomedial tears elicit incomplete neointimal healing without restoration of elastic laminae.

### Differentiated smooth muscle cells are the predominant cell type of the tear neointima.

**Figure 4:**
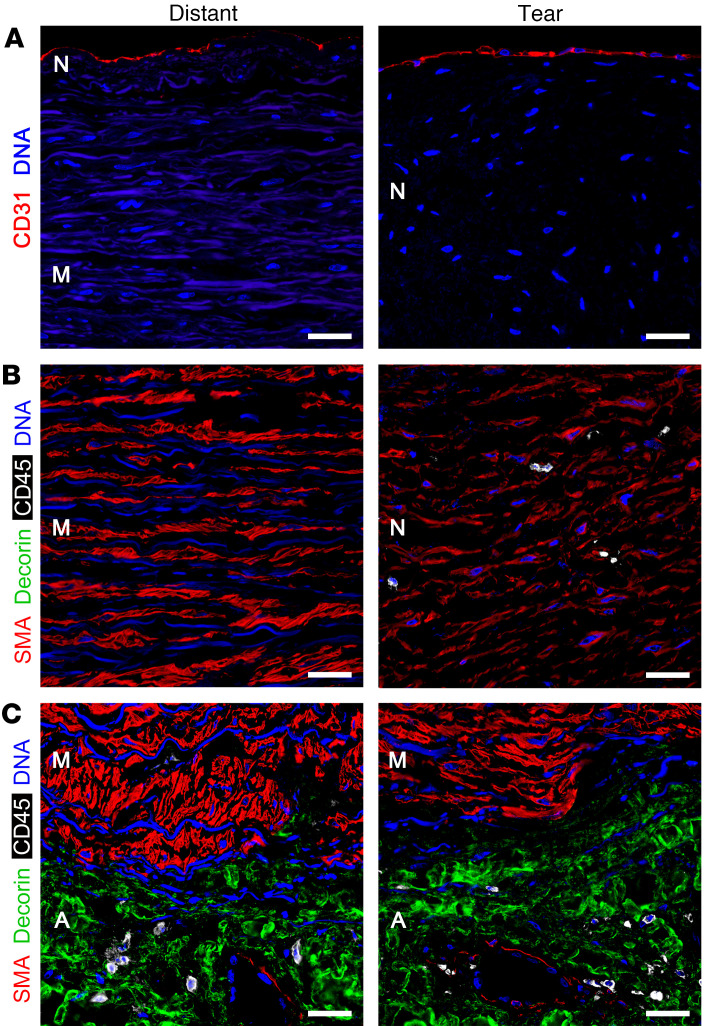
Cell types in intimomedial tears. Aortas with intimomedial tears were analyzed by immunofluorescence confocal microscopy. (**A**) The endothelial cell marker CD31 (red) is detected on the luminal surface of both distant aorta and tear neointima without evidence of mural neovascularization. (**B**) The SMC marker SMA (red) is detected in most cells of the distant media and tear neointima. Distant media cells are spindle shaped and circumferentially oriented, whereas tear neointima cells are smaller and irregularly shaped. Scattered CD45^+^ leukocytes (white) are detected in the tear neointima, but not distant media. (**C**) SMA^+^ SMCs of uniform size and shape are the exclusive constituents of the distant media and tear base, while the fibroblast product decorin (green) is restricted to the adventitia where single layers of SMCs support occasional microvessels. In addition to DAPI staining nucleic DNA (blue) indicating cell density and orientation, autofluorescence (less intense blue) of elastic laminae is detected in the distant media and tear base, but not the tear neointima. Orientation: internal aspect above, external aspect below. N, neointima; M, media; A, adventitia. Scale bars: 25 μm.

The identity of cells forming the tear neointima was investigated by immunofluorescence confocal microscopy. CD31^+^ endothelial cells covered the luminal aspect of intimomedial tears and distant aorta, without evidence of mural neovascularization (Figure 4A). Almost all cells within the tear neointima expressed α-smooth muscle actin (SMA), suggesting a smooth muscle cell (SMC) lineage (Figure 4B). These cells appeared smaller in size and more irregular in shape and orientation than SMCs of the distant media. CD45^+^ leukocytes were scattered within the neointima and rare in the distant media, while decorin-producing fibroblasts were restricted to the adventitia (Figure 4C).

**Figure 5:**
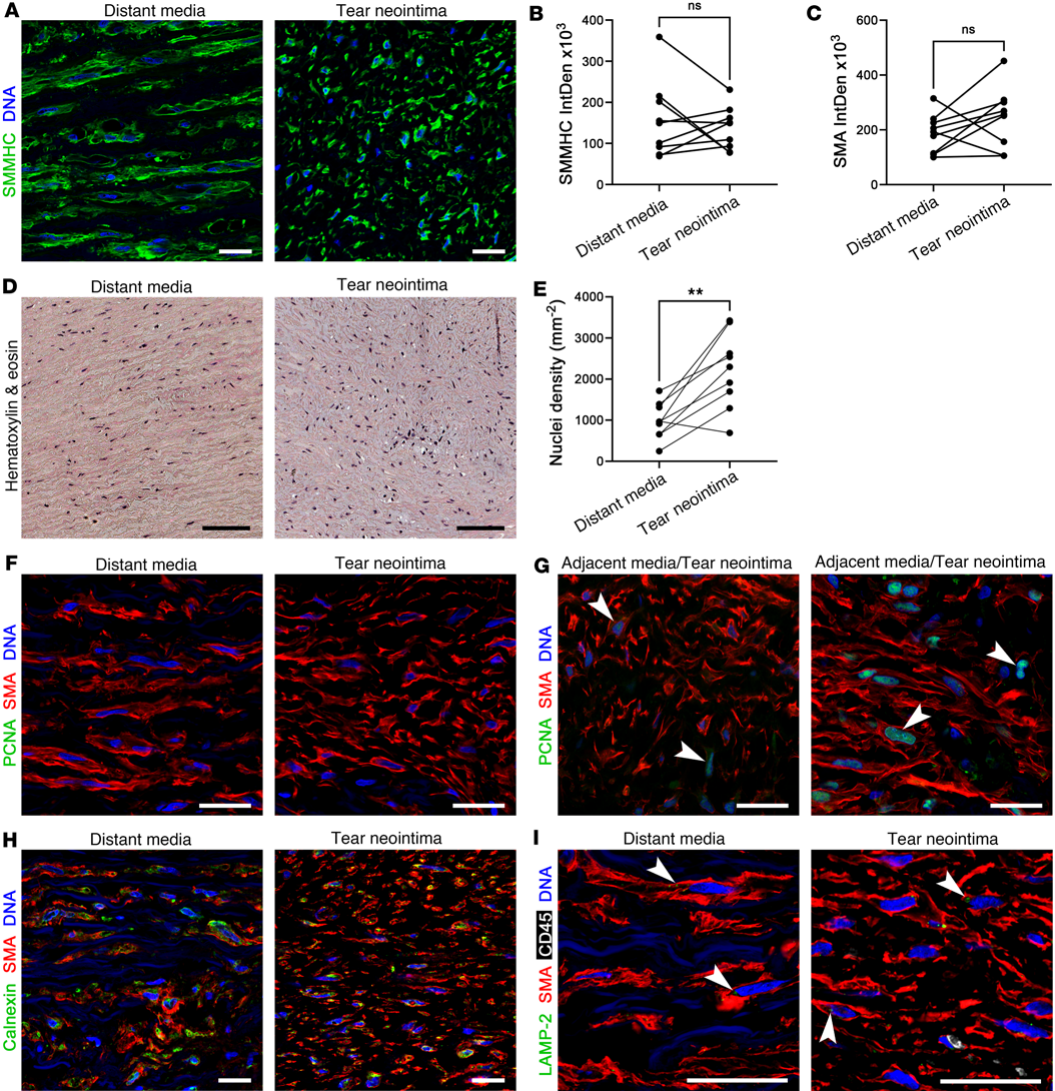
SMC phenotype in intimomedial tears. Aortas with intimomedial tears were analyzed by histology and immunofluorescence confocal microscopy. (**A**) The SMC contractile protein SMMHC (green) is detected in distant media and tear neointima cells with (**B**) similar signal intensity (integrated density, IntDen). (**C**) Expression of SMA (red, panels **F**–**I**) is also of similar intensity. (**D**) Hematoxylin and eosin staining identifies nuclei (purple) with (**E**) increased density in tear neointima. (**F**) PCNA (green, arrows) is rarely detected in SMCs, (**G**) except in a few cells at the interface of adjacent media and tear neointima (left panel) — including an atypical lesion with marked SMC proliferation (right panel). Comparable expression of (**H**) the endoplasmic reticulum marker calnexin (green) and (**I**) the lysosome marker LAMP-2 (green, arrows) in SMA^+^ SMCs. Black scale bars: 100 μm; white scale bars: 25 μm. Data are means, with lines connecting values from individual specimens (*n* = 9). NS, not significant. **P < 0.01 by paired, 2-tailed *t* test.

The phenotype of lesional SMCs was further examined. Cells within the tear neointima expressed an additional contractile protein, smooth muscle myosin heavy chain (SMMH), at similar levels to that of distant media cells, corroborating their identity as SMCs and signifying a contractile phenotype (Figure 5, A and B). Quantification of SMA also showed comparable levels (Figure 5C). Enumeration of nuclei in histological stains revealed an increased density of cells in the tear neointima (Figure 5, D and E). Proliferating cell nuclear antigen (PCNA) was not detected, except in a few SMCs at the interface of adjacent media and tear neointima, suggesting a gradient of dividing cells from intact tissue (Figure 5, F and G). PCNA^+^ SMCs did not express CD34 to suggest stem cell or vascular-associated progenitor cell populations (Supplemental Figure 3). Expression of calnexin and lysosomal-associated membrane protein 2 (LAMP-2), markers of the endoplasmic reticulum and lysosomes, respectively, did not differ among SMCs of the tear neointima and distant media (Figure 5, H and I). Moreover, there was similar distribution of the mitochondrial regulators, peroxisome proliferator-activated receptor γ coactivator 1-α (PGC-1α) and mitochondrial transcription factor A (TFAM) among lesional versus medial SMCs (Supplemental Figure 4). Together, these data confirm an SMC identity of cells within the tear neointima displaying a contractile phenotype, with homeostatic levels of synthetic, degradative, and bioenergetic organelles despite evidence of cellular proliferation and altered cell morphology.

### Tear neointima is characterized by dense fibrosis and immature elastic fibers, while tear bases of wide lesions have greater fragmentation of elastic laminae.

**Figure 6:**
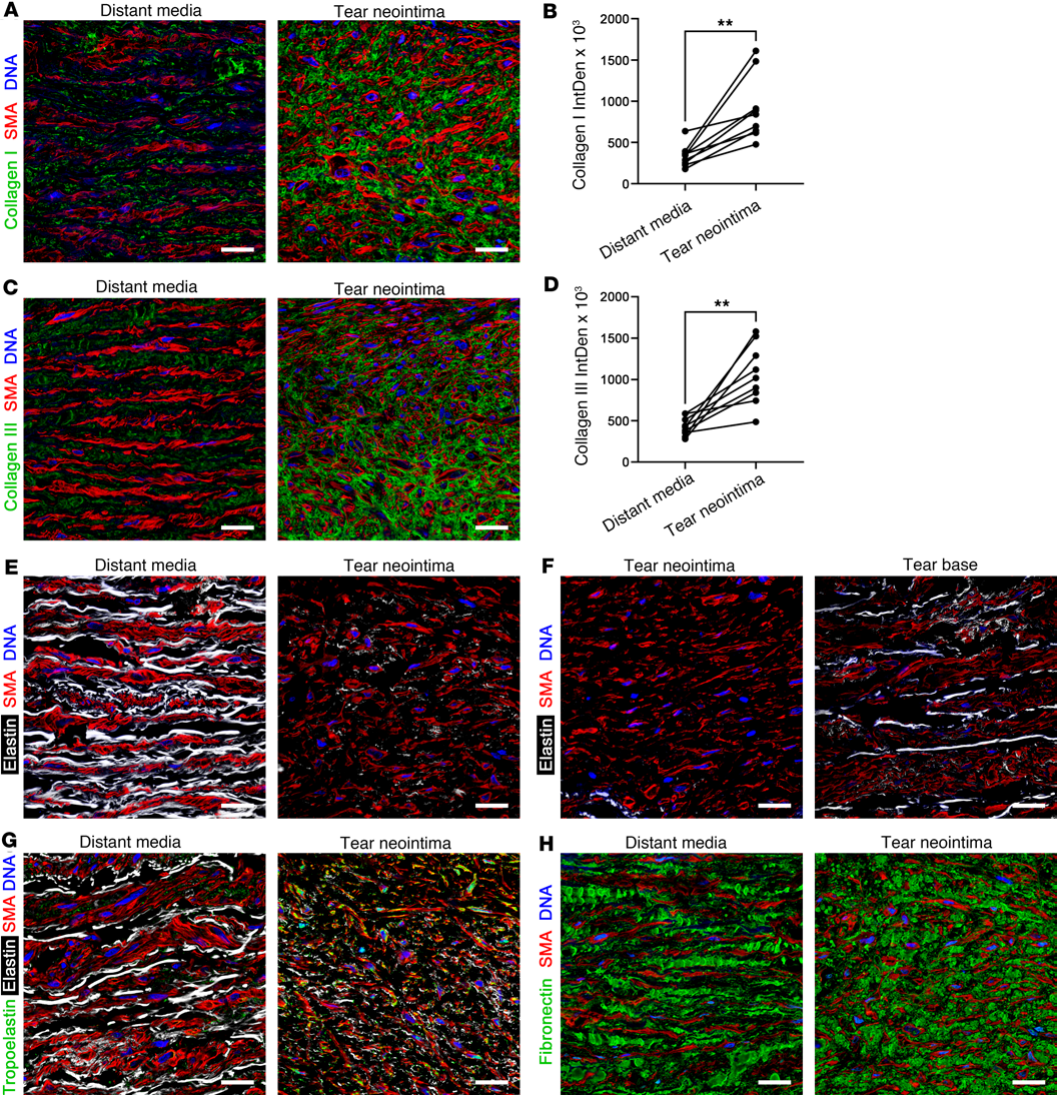
ECM changes in intimomedial tears. Aortas with intimomedial tears were analyzed by immunofluorescence confocal microscopy. (**A**) Increased type I collagen (green) surrounding SMA^+^ SMCs in tear neointima versus parallel bundles in distant media with (**B**) greater signal intensity (integrated density, IntDen). (**C**) Similarly, increased type III collagen (green) in tear neointima with (**D**) greater signal intensity. (**E**) Less elastin (white) in tear neointima with punctate appearance or as short, thin fibers versus parallel thick elastic laminae with extensions of intralaminar elastic fibers in the distant media. (**F**) Absent elastin in other areas of tear neointima, while elastic laminae are fragmented with fewer intralaminar elastic fibers in some tear bases. (**G**) Increased elastin precursor tropoelastin (green) in tear neointima compared with distant media. (**H**) Similar levels of fibronectin (green), although with pericellular pattern in tear neointima versus parallel arrangement in distant media. Scale bars: 25 μm. Data are means, with lines connecting values from individual specimens (*n* = 9). **P < 0.01 by paired, 2-tailed *t* test.

The ECM of intimomedial tears was characterized by histology and immunofluorescence confocal microscopy. Increased collagen and absent elastic laminae in the tear neointima were confirmed in Sirius red and Verhoeff–Van Gieson stains, respectively (Supplemental Figure 5, A and B). Specific antibodies against type I and III collagen demonstrated increased expression in the tear neointima that surrounded SMCs instead of bundles arranged in parallel layers, as in the distant media (Figure 6, A–D). Visualization of elastin by Alexa Fluor 633 hydrazide dye revealed a pericellular punctate distribution with occasional thin, short fibers in the tear neointima, suggesting newly synthesized protein, unlike thick elastic laminae with extensions of intralaminar elastic fibers in the distant media (Figure 6E). The findings were heterogeneous, with other areas of the tear neointima almost devoid of elastin, while some tear bases contained fragmented elastic laminae and few intralaminar elastic fibers (Figure 6F). An antibody more selective for the soluble precursor, tropoelastin, than insoluble elastin confirmed greater detection of immature elastin in tear neointima than distant media (Figure 6G). Analysis of multiple lesions from the aorta with subacute injury showed that tear bases of narrow defects had relatively intact elastic laminae, but tear bases of wide lesions had considerable disruption of elastic laminae with fragmentation extending into the adjacent outer media (Supplemental Figure 5, C and D). In contrast with collagen and elastin abnormalities, the proteoglycan fibronectin was similarly detected in tear neointima and distant media (Figure 6H). Thus, the tear neointima resembles a scar with marked fibrosis and loss of normal elastic fiber architecture, while stretching of tear bases in wide lesions fragments the elastic laminae.

### Intimomedial tears contain fibrin clots, but no extravasation of plasma or erythrocytes nor accumulation of lipid.

**Figure 7:**
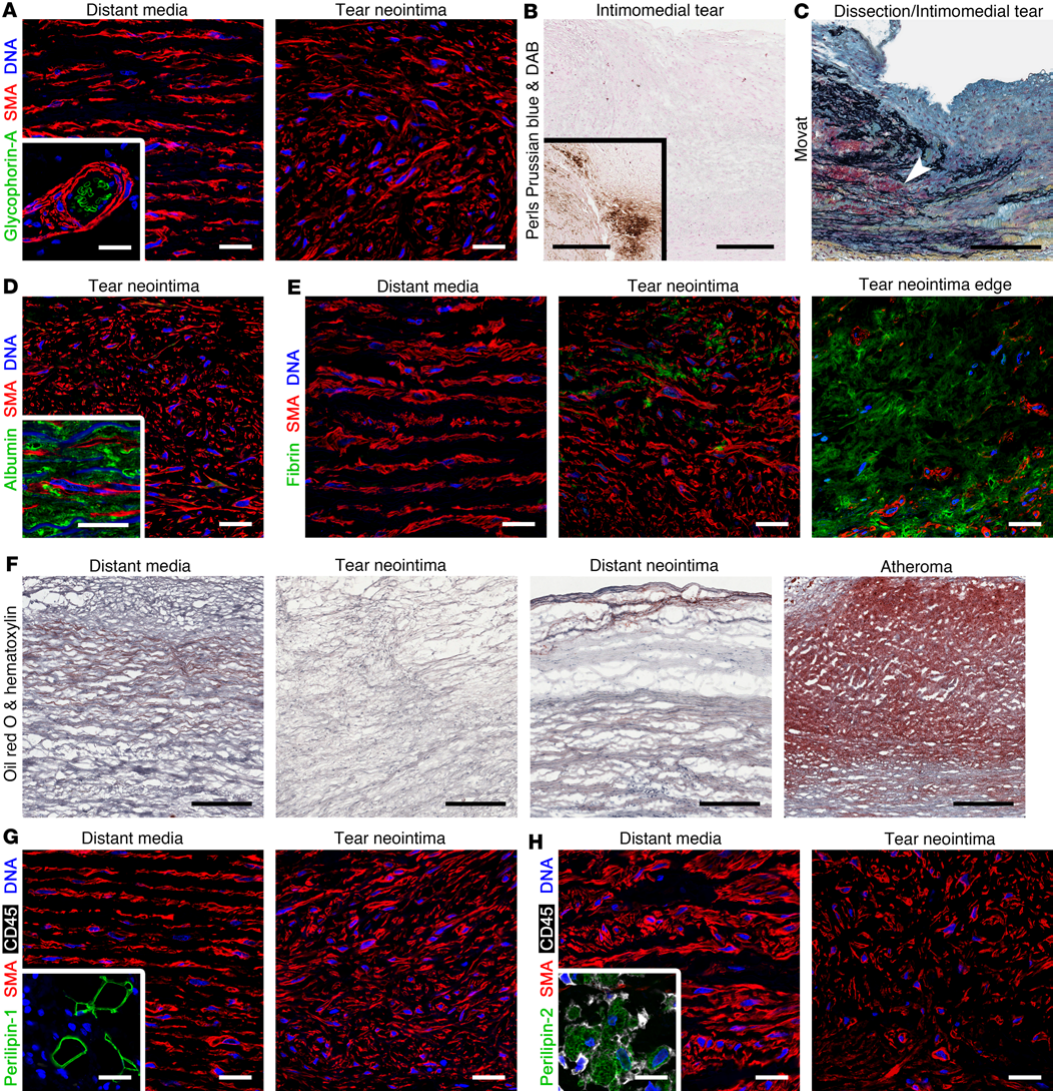
Interstitial changes in intimomedial tears. Aortas were analyzed by histology and immunofluorescence confocal microscopy. (**A**) Erythrocyte marker glycophorin-A (green color) is not detected in distant media or tear neointima, although red blood cells are seen in adventitial microvessels (inset). (**B**) Absent Perls Prussian blue/diaminobenzidine (DAB) staining for iron (brown) in tear neointima, but strong staining in positive control of chronic aortic dissection (inset). (**C**) Medial extravasation of red blood cells (red) from nearby dissection is restricted (arrow) by fibrotic neointima of intimomedial tear. (**D**) Albumin (green) is not detected in tear neointima, but is found in positive control of acute aortic dissection (inset). (**E**) Fibrin (green) is detected in tear neointima, but not distant media, with organization into dense structures at tear edges. (**F**) Oil red O staining for neutral lipid (red) is absent in distant media and tear neointima, weak in neointima overlying distant media, and strong in aortic atheroma without intimomedial tear. (**G**) Perilipin-1 (green) and (**H**) perilipin-2 (green) are not detected in SMCs of distant media or tear neointima but are present in adventitial adipocytes and plaque foam cells (insets), respectively. Black scale bars: 250 μm; white scale bars: 25 μm.

The interstitium of the tear neointima was analyzed for anomalous constituents. Consistent with a nonhemorrhagic macroscopic appearance, there was no neointimal or medial extravasation of glycophorin-A^+^ red blood cells (Figure 7A). Furthermore, there was no histological signs of iron accumulation to indicate prior degradation of erythrocyte-derived hemoglobin (Figure 7B). Intimomedial tears appeared resistant to mural hemorrhage, as in 1 specimen with acute dissection contiguous to a healed intimomedial tear, red blood cells infiltrated the distant and adjacent media but did not extend into the fibrotic tear neointima (Figure 7C). Furthermore, the plasma protein albumin was not detected within neointimal lesions (Figure 7D). Fibrin, however, was detected within the tear neointima and was denser and more organized in areas close to the edges with adjacent media (Figure 7E). In keeping with an absence of visible atheromatous plaques, neutral lipids did not accumulate in the tear neointima or distant media, but were detectable in neointima overlying the distant media and substantial accumulation was evident in plaques of atherosclerotic aortas without intimomedial tears (Figure 7F). Additionally, perilipin-1 and perilipin-2, which coat lipid droplets in adipocytes and foam cells, respectively, were not expressed in SMCs of tear neointima nor distant media (Figure 7, G and H). These data reveal incorporation of thrombus within healed intimomedial tears but no evidence of mural hemorrhage or atheroma formation.

## Discussion

Tears through the intima and partially into the subjacent media represent a minimal injury of the aorta. Correlation with aortic dilatation suggests that increased wall stresses and/or medial degeneration are etiological factors, while location of lesions within the proximal aorta and correlation with aortic valve regurgitation implicate axial wall stress (9). Uncomplicated intimomedial tears heal via neointimal SMCs producing a collagen-rich matrix, resulting in scars without significant inflammation or lipid accumulation. Although incidental findings of chronic lesions imply a benign course, others have documented that limited tears, albeit complicated by mural hematomas, may rapidly progress to rupture or dissection with fatal consequences (7). The relationship and differences between intimomedial tear and rupture or dissection is important in understanding structural failure of the aortic wall and informing its clinical management.

The postmortem and surgical studies that first defined limited tears of the aorta described intimal defects without signs of rupture or dissection (2, 3). Pathological findings specified “no significant intramedial dissection” and “without separation of the medial layers,” but the series of cases also included “limited intramedial dissection of blood” and “a minimal amount of blood in the dissected aortic wall.” Furthermore, clinical presentation varied from incidentally found lesions to bloody cardiac tamponade, the latter implying at least contained rupture with restricted egress of blood. Thus, both uncomplicated intimomedial tears and those with some degree of dissection or rupture were categorized together. Subsequent imaging studies and numerous case reports focused on complex limited tears associated with varying mural hematoma and medial dissection (even exceeding 20 mm), contained and free rupture, bloody pericardial effusion and cardiac tamponade, as well as death (7, 8, 10–20). Nonetheless, several histological studies unequivocally described healed intimomedial tears with no evidence of dissection or rupture (21–24). These diverse observations led to mural lesions that are not classic extensive dissection or free rupture, collectively referred to as limited tears. Relatively uncomplicated intimomedial tears were identified in the current study, with the tear edge of a subacute lesion undermined by less than 3 mm signifying a nonhealed short dissection, and fibrosis extending less than 1 mm into the adjacent media in 2 of 8 chronic lesions signifying healed minimal dissections. Thus, we propose that uncomplicated partial mural tears with minimal medial delamination of less than a few millimeters (i.e., unlikely to be identified by CT scan or TEE) be considered as intimomedial tears, while lesions with moderate medial delamination of greater than a few millimeters but less than a few centimeters (i.e., likely to be identified as intimomedial flap and intramural hematoma by CT scan or TEE) be considered as focal dissection and intimomedial tears extending through the media, with subadventitial hematoma considered contained rupture.

**Figure 8:**
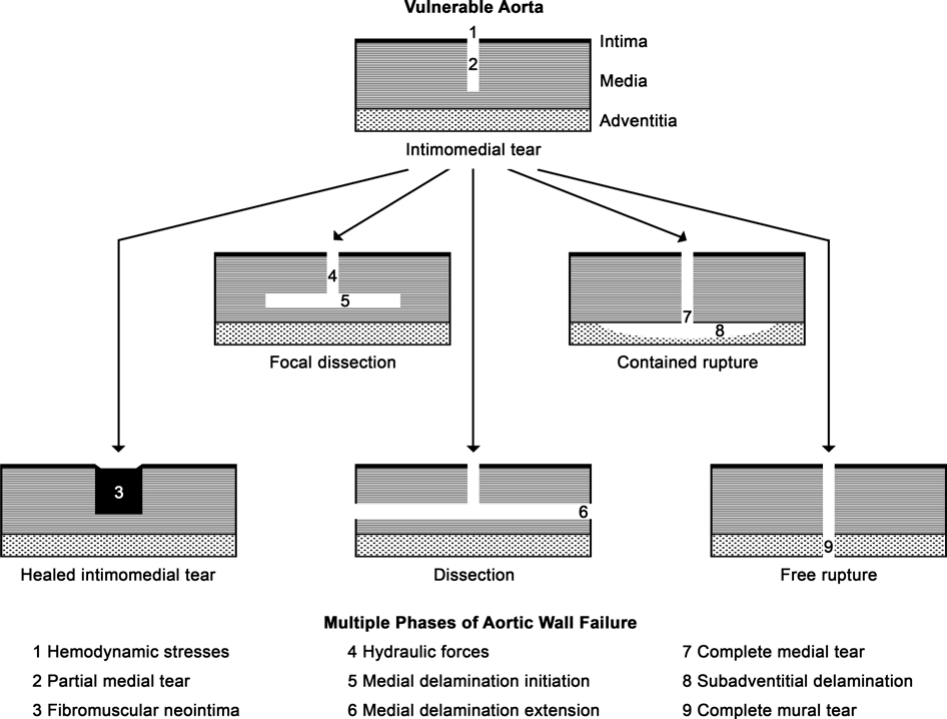
Multiphasic model of aortic wall failure. Mismatch between wall stress and wall strength may lead to tears of the aorta involving the intima and part of the subjacent media (intimomedial tear). The lesions may heal without complication (healed intimomedial tear) or extend, suddenly or gradually, by separating the layers of the media in axial and circumferential planes as dissection or completion of tears through the media in a radial direction as rupture. Medial delamination may uncommonly be circumscribed (focal dissection) or typically extensive (classic dissection, or simply dissection). Bleeding through transmedial tears may be restricted by adventitia and fibrotic perivascular tissue (contained rupture) or result in exsanguination through transmural extension (free rupture). Each phase of wall failure (labeled 1–9) likely requires distinct biomechanical forces or cellular and ECM vulnerabilities, implying multiple mechanisms and therapeutic targets. Thus, a continuum of aortic injury is created by varying combinations of defects.

Recognition of intimomedial tears as the least severe type of aortic injury implies a multiphasic model of aortic wall failure in which partial tears of the intima and media heal or extend as more complex lesions of dissection or rupture, depending on resilience factors (23). We speculate that each phase of wall failure is determined by distinct biomechanical forces or cellular and ECM vulnerabilities and varying combinations of defects contribute to a continuum of aortic injury (Figure 8). Injuries include the occurrence and repair of intimomedial tears by (i) biaxial wall stresses exceeding wall strength, resulting in (ii) radial tears through the intima and part of the subjacent media that (iii) partially heal by fibromuscular neointima. Alternatively, intimomedial tears may progress to dissection by (iv) blood hydraulic forces within the radial defect, (v) initiating delamination of the media that uncommonly remain focal, but (vi) usually extend considerably in axial and circumferential planes. In other cases, intimomedial tears may progress to rupture by (vii) tear completion through the outer media, allowing (viii) bleeding that is contained by intact adventitia, although it may strip from the external elastic lamina or (ix) exsanguination via torn adventitia. The progression of intimomedial tears to dissection or rupture can occur rapidly or gradually, dissections may rupture from secondary structural deterioration, and uncommonly nonlethal dissection and rupture can heal by similar processes as intimomedial tears. While healed intimomedial tears, extensive (classic) dissection, and free rupture have distinct phenotypes representing extremes of the disease spectrum, focal dissection with discrete mural hematomas and contained rupture with conspicuous mural hematomas have intermediate phenotypes often misdiagnosed. Segregation of phenotypes is problematic if intimomedial tear is classified as a type of dissection (3) instead of categorizing intimomedial tear, dissection, and rupture as types of mural tears (2).

Observations that intimomedial tears may heal without apparent sequelae justifies separate classification. In contrast, focal dissection associates with intimomedial flaps and even saccular aneurysms, prompting therapeutic intervention (2, 8, 25–27), whereas contained rupture is generally accepted as an indication for urgent intervention in any segment of the aorta (28). Notably, we did not find fibrotic neointima extending into the distant media or the absence of a tear base to suggest healing of focal dissection or contained rupture, respectively. We do not exclude partial healing of some complex tears, as a few patients with dissection of the ascending aorta survive without repair (29). Criteria to prescribe the management of spontaneous intimomedial tears have not been proposed. Such criteria have been developed for traumatic aortic injury based on CT scan findings. In blunt trauma of the descending thoracic aorta (segment with intermediate risk from tear complications), isolated intimomedial tears are managed nonoperatively, whereas mural hematomas of any size and all contained ruptures are repaired by open or endovascular means (30). In blunt trauma of the abdominal aorta (segment with least risk from tear complications), limited tears with defects or thrombus less than 10 mm are managed nonoperatively, large intimal flaps with defects or thrombus of 10 mm or greater are selectively managed depending on lesion progression, and contained or free rupture are managed operatively (31, 32). Our findings suggests that nontraumatic intimomedial tears of the ascending aorta (segment with greatest risk from tear complications) may also be managed nonoperatively, whereas focal dissections greater than a few millimeters (sufficient to be detected by imaging studies) and any contained ruptures should be managed operatively. Since a paucity of symptoms and the resolution of imaging studies likely preclude diagnosis at disease onset, the recommendations are relevant for incidental findings of subtle mural abnormalities by CT scan and TEE or discovery of healed intimomedial tears during surgery where replacement of the ascending aorta is not planned, e.g., during isolated aortic valve replacement.

Identification of intimomedial tears by inspection of operative specimens and confirmation by histology is not an accurate indicator of disease incidence. During the study period, 2 cases were encountered with sudden onset aortic valve regurgitation from acute intimomedial tears near valve commissures and the lesions were not excised but incorporated within repair of the aorta and valve resuspension. Additionally, several small scars near aortic valve commissures or coronary artery ostia were not excised, as the involved tissue was required for valve-sparing aortic root replacement and reimplantation of coronary artery buttons. The lack of submillimeter lesions or those involving less than 40% of the media suggests that small tears may have been missed. Current imaging technology, however, is unlikely to supplant macroscopic examination in identifying intimomedial tears. Both lesions detected by retrospective analysis of CT scans had mural attenuation of 0.5–1 mm compared with the adjacent and distant media, whereas 6 of 7 intimomedial tears not identified by CT scan had less than 0.5 mm wall thinning. Of relevance, spatial resolution of cardiac-gated, contrast-enhanced CT scans is approximately 0.5 mm (33). Targeted biopsies are key to histological diagnosis of intimomedial tears in diseased aortas and requires discrimination among numerous aortic fragments available to pathologists after surgery. Alternatively, quantification of intimomedial tears in experimental studies is accomplished by continuous sections of the aorta (34). Although challenging for larger human specimens, serial sections of seemingly normal tissue may be necessary to identify possible microscopic lesions in nondilated aortas. Our analyses of intimomedial tears are limited to histology and immunofluorescence microscopy that informed about tissue architecture as well as localization and quantification of proteins. Bulk measurements (e.g., Western blotting, quantitative RT-PCR) are not appropriate for small, partial-thickness lesions and methods for individual cells without spatial context (e.g., flow cytometry, single-cell RNA-seq) are not suitable because of limited and skewed cell isolation. Attempts at spatial transcriptomics were unsuccessful, with very few genes detected in formalin-fixed, paraffin-embedded aortic tissue.

Our translational study was motivated by prior research findings. We, and others, have described intimomedial tears of the murine aorta after induction of hypertension (34–36). By microscopy, isolated tears through the intima and inner media are distinct from aortic dissection and rupture. Pharmacological inhibition of mTOR signaling prevented contained ruptures, but predisposed to medial dissection of the abdominal aorta in hyperlipidemic mice (36). It remains unknown which cellular and ECM defects contribute to transmedial rupture in a radial direction versus medial delamination along axial and circumferential planes. The incidental finding of healed intimomedial tears, however, establishes that some compromised aortas can resist tear propagation to rupture or dissection. In the current study, distraction of tear edges from less than 1 mm in acute entry tears up to 20 mm in chronic intimomedial tears with marked fragmentation of elastic laminae in the underlying tear base provides a mechanism for rapid aortic enlargement via stretching of lesions. Conversely, documentation of accelerated aortic growth during radiological monitoring of patients with dilated aortas should raise a suspicion for intimomedial tears.

Microscopy reveals differences in neointimas associated with intimomedial tears compared with atherosclerotic plaques. The tissue-filling intimomedial defects may be considered neomedia in the absence of an intact internal elastic lamina that delineates the inner vessel wall layers. We prefer neointima, since new tissue forms internal to residual media of the tear base and there is precedence for the terminology (2). SMCs are the near-exclusive cell type constituting the reparative neointima besides few, if any, leukocytes (cf. Figure 4B with few leukocytes and Figure 7, F and G with no leukocytes). Surprisingly, SMCs of the tear neointima exhibit a differentiated phenotype, expressing similar SMA and SMMHC to that of medial SMCs. While SMCs in atherosclerotic plaques are typically dedifferentiated, SMCs in fibrotic atherosclerotic plaques of young patients may also display a contractile phenotype (37). Tear neointimal SMCs synthesize abundant type I and III collagen and some immature, small elastic fibers but elastic laminae are not restored. It is unknown whether the atypical ECM contributes to the smaller size and disorganized orientation of SMCs. In one case with dissection contiguous to a healed intimomedial tear, extravasated red blood cells did not track into the fibrotic tear neointima, suggesting that fibrillar collagen rather than SMCs and elastic laminae prevents progression of hemorrhage. This is not unlike the limited bleeding from penetrating atherosclerotic ulcers where medial fibrosis is thought to hamper dissection despite SMC and elastic laminae abnormalities (38). Although chronic intimomedial tears may result in wall thinning and focal mural bulging, the tear neointima is sufficiently robust to prevent dissection or rupture. An exception may be multiple crater-like lesions in which a case of delayed rupture was reported (39).

It may be considered unexpected that atherosclerosis is not linked to intimomedial tears, as intimal plaques of the aorta can rupture and their presence is associated with atrophy of the underlying media (40, 41). Nevertheless, the observation that atherosclerosis is uncommon in relation to ascending aorta tears is established (2, 42). On the other hand, it may be considered surprising that intimomedial tears do not lead to atherosclerosis. The classic opposing hypotheses for atherosclerosis formation by Rokitansky and Virchow invoked thrombus incorporation and response to injury, respectively (43). While the former concept has limited experimental support and lost popularity (44), the latter concept prevailed with modern investigators elucidating myriad pathogenetic mechanisms (45). Mural fibrin within tear neointima is unlike thrombosed arteries that have recannulated and likely represents persistence of original thrombus in acute tears that remain at the edges as the healing lesion stretches. Notably, neither persistent thrombus nor SMC responses to physical injury predispose to leukocytic infiltrates or lipid accumulation even though most patients with intimomedial tears in our study had risk factors for cardiovascular disease and several had atherosclerosis in other locations of the aorta or other arteries. Additionally, atherosclerotic plaques were not noted in previous histological reports of healed intimomedial tears (21–24). Absent signs of atherosclerosis in intimomedial tears may be due to insufficient disease duration, although it is likely that healed lesions develop over many months to several years from initial injury within a time frame for progression of conventional atherosclerosis or accelerated onset of in-stent restenosis (46, 47). Instead of causing disease, tear neointimas represent a reparative role for SMC proliferation and fibrosis consistent with Russel Ross’s proposal of a defense mechanism gone awry in atherosclerosis (48).

In conclusion, intimomedial tears are largely a silent disorder, heal spontaneously, and are not an indication for therapeutic intervention. The condition, unless complicated by focal dissection or contained rupture, is likely of little relevance in patient management and may explain why limited tears are not included in recent reviews of acute aortic syndrome and clinical practice guidelines for the diagnosis and management of aortic disease (1, 28), whereas these lesions were previously recognized by the authors (5, 49). Characterizing minor complications, however, is important in understanding pathogenetic mechanisms that can progress to catastrophic disorders or differentiate adaptive from pathological responses. Therefore, continued attention to, and greater recognition of, intimomedial tears is warranted.

## Methods

### Sex as a biological variable.

Both sexes were included in the study. There were no significant differences in patients with and without intimomedial tears with respect to sex, age, race, or ethnicity.

### Patients and aortic specimens.

Ascending aortic tissue was obtained from 108 patients who underwent thoracic aortic surgery during an 18-month period (July 2021 to January 2023) at Yale New Haven Hospital. We adhered to STROBE statement reporting guidelines for observational studies (https://www.strobe-statement.org/). The patients were not from consecutive operations, as inclusion depended on the availability of research personnel. Specimens were procured within the operating room to enable precise anatomical location and orientation. Excised aortas were inspected for intimal defects in coordination with the surgeon’s observations. A custom calculator (https://medicine.yale.edu/surgery/cardio/research/) was used to normalize aortic diameter by age, sex, height, and weight to determine expected diameter, fold increase, and *z* score (50). Aortas were defined as nondilated (≤2 SD of expected diameter), ectasia (>2 SD but ≤1.5-fold expected diameter), and aneurysm (>1.5-fold expected diameter). Genetic testing was by Yale’s Department of Genetics using whole-exosome sequencing and variants were reported for 20 or more genes related to thoracic aortic aneurysms and dissection (51). Of 9 patients with intimomedial tears, all underwent genetic testing: 6 had no clinically significant variants, 2 had variants of uncertain significance, and 1 had a likely pathogenic variant (*FBN1*: c.7956T>A). Of 97 patients without intimomedial tears, 1 had a clinical diagnosis of Marfan syndrome without genetic testing at our institution and 62 underwent genetic testing: 38 had no clinically significant variants, 20 had variants of uncertain significance, 3 had likely pathogenic variants (*SMAD3*: c.364G>A, *MYH11*: c.3749T>C, *FBN1*: c.6919T>C), and 1 had a pathogenic variant (*FBN1*: c.493C>T).

### Imaging studies.

CT angiography was performed using Somatom Force (Siemens) or Revolution HD, Discovery CT750 HD, Optima CT660, and LightSpeed VCT (GE HealthCare) scanners. Three-dimensional volume rendering and virtual fly-through processing was performed with Visage (version 7.1.17, Visage Imaging) and Vitrea (version 7.15.6, Canon Medical) software, respectively. TEE was performed using an Epiq 7c Ultrasound System with an X5-1 Matrix Array Probe (Philips).

### Histology.

Transverse sections of aortas were fixed in 4% paraformaldehyde overnight at 4°C and embedded in paraffin. Alternatively, selected specimens were embedded in optimal cutting temperature compound and frozen at –80°C to avoid removal of neutral lipids. Tissue blocks were sectioned at 5 μm thickness and Movat’s pentachrome, hematoxylin and eosin, Verhoeff–Van Gieson, Sirius red, Perls Prussian blue with diaminobenzidine enhancement, and Oil red O stainings were performed by Yale’s Research Histology Laboratory using standard techniques.

### Histomorphometry.

Stained slides were digitized using an Aperio AT2 scanner (Leica). The thickness of vessel wall layers was measured using QuPath software (https://qupath.github.io) from Verhoeff–Van Gieson–stained images. Medial and neointimal thickness was averaged from 3 measurements in the distant aorta (more than several millimeters from tears), adjacent aorta (within a few millimeters of tears), and aortic tears (encompassing intimomedial tears and underlying base). Alternatively, nuclear density was calculated using ImageJ software (http://imagej.net) from hematoxylin and eosin–stained images at ×200 magnification. Images from 4 representative areas (0.12 mm^2^) were split into the red channel where stronger contrast exists between nuclei and surrounding elements and the threshold function was adjusted to encompass nuclei. Counts of particles of specified size and circularity were obtained using the Analyze Particles command and nuclear density was calculated for the distant media and tear neointima.

### Immunofluorescence confocal microscopy.

Formalin-fixed, paraffin-embedded tissue blocks were sectioned at 5 μm. The slides were serially dewaxed in xylene and rehydrated in a series of graded alcohol and then water. Heat-mediated antigen retrieval was performed (H-3300-250, Vector Laboratories). Sections were incubated overnight at 4°C with antibodies against SMA (1-9760-82, Thermo Fisher Scientific or ab5694, Abcam), SMMHC (53-6400-82, Thermo Fisher Scientific), CD31 (ab9498, Abcam), CD45 (LS-B14248-300, Lifespan Biosciences), decorin (HPA003315, Atlas Antibodies), PCNA (13110S, Cell Signaling Technology), CD34 (MA5-16924, Thermo Fisher Scientific), calnexin (ab22595, Abcam), LAMP-2 (ab25631, Abcam), PGC-1α (66369-1-Ig, Proteintech), TFAM (22586-1-AP, Proteintech), collagen I (72026, Cell Signaling Technology), collagen III (22734-1-AP, Proteintech), tropoelastin (PR398, Elastin Products Company), fibronectin (15613-1-AP, Proteintech), glycophorin-A (13-9987-82, Thermo Fisher Scientific), albumin (16475-1-AP, Proteintech), fibrin (55169, Cappel), perilipin-1 (9349, Cell Signaling Technology), and perilipin-2 (MAB76341, R&D Systems). Fluorescein-labeled *Ulex europaeus* agglutinin I was purchased from Vector Laboratories (FL-1061). Detection of unconjugated primary antibodies was visualized with Alexa Fluor 488–, 568–, or 647–conjugated IgG (Invitrogen). Elastic fibers were visualized with Alexa Fluor 633 hydrazide (A30634, Thermo Fisher Scientific) and nucleic DNA with DAPI (D1306, Thermo Fisher Scientific). Sections were mounted with ProLong Gold Antifade (P36984, Thermo Fisher Scientific). The slides were scanned using a Stellaris 8 Falcon confocal microscope with LAS X software (Leica) for image acquisition.

### Fluorescence intensity.

Images of SMMHC, SMA, collagen I, and collagen III expression were acquired in single-color channels at ×400 magnification from representative areas of distant media and tear neointima in 9 specimens. Fluorescence intensity was quantified using ImageJ software. Pixel size was calibrated, the automated threshold function was set to isolate signal from background, and integrated density (product of area and mean gray value) was measured.

### Statistics.

Continuous data are expressed as mean ± SD and categorical data were tabulated as counts and percentages. Comparisons of continuous variables were performed using unpaired *t* test for 2 independent groups, paired *t* test for 2 related measurements in the same group, and repeated measures ANOVA with Tukey’s multiple-comparison test for more than 2 related measurements in the same group. Categorical data of 2 independent groups were compared using Fisher’s exact test. Probability values were 2-tailed and a *P* value of less than 0.05 was considered to indicate statistical significance. Statistical analyses were performed using Prism (version 9.5.0, GraphPad Software).

### Study approval.

Research protocols to obtain aortic tissue and clinical information from patients undergoing thoracic aortic surgery were approved by the Institutional Review Boards of Yale University with a waiver for consent. The procedures followed were in accordance with institutional guidelines.

### Data availability.

All data are included in the main manuscript and supplemental materials. Values for all data points in graphs are reported in the online Supporting Data Values file. Additional deidentified data may be available on written request.

## Author contributions

AHMH, GT, and RA designed the study. AHMH conducted experiments and acquired data. AHMH, DJH, GT, and RA analyzed and interpreted data. GT and RA supervised the work. AHMH, DJH, PV, GT, and RA wrote and edited the manuscript. The 2 senior authors (GT and RA) equally shared supervision of the work.

## Supplementary Material

Supplemental data

ICMJE disclosure forms

Supporting data values

## Figures and Tables

**Table 1:**
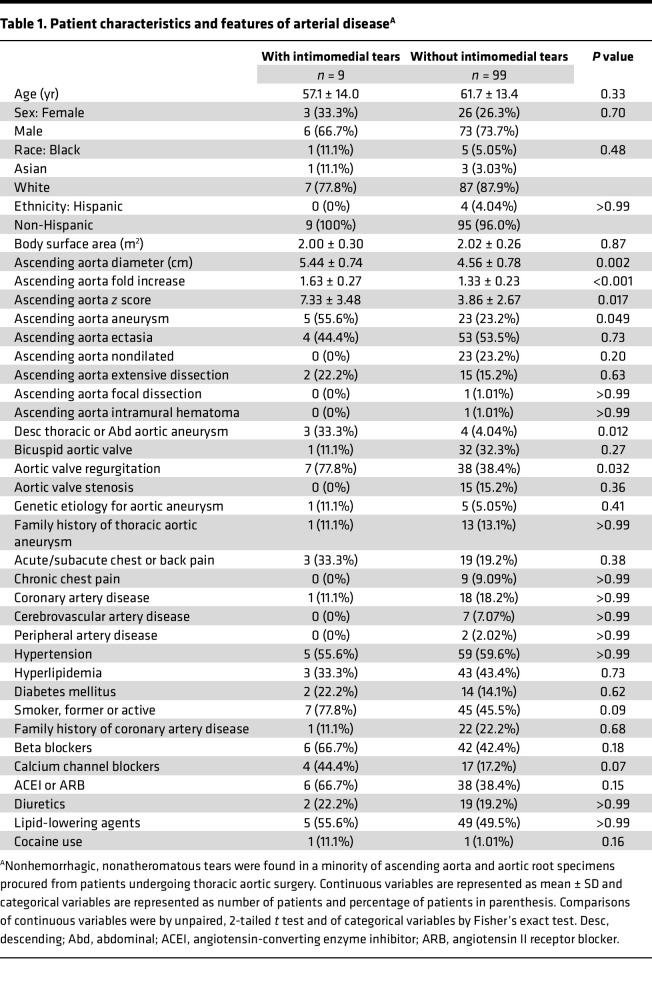
Patient characteristics and features of arterial disease^A^

## Version 1 04/09/2024

In-Press Preview

## Version 2 05/08/2024

Electronic publication

## References

[1] Vilacosta I (2021). Acute aortic syndrome revisited: JACC state-of-the-art review. J Am Coll Cardiol.

[2] Murray CA, Edwards JE (1973). Spontaneous laceration of ascending aorta. Circulation.

[3] Svensson LG (1999). Intimal tear without hematoma: an important variant of aortic dissection that can elude current imaging techniques. Circulation.

[4] Erbel R (2001). Diagnosis and management of aortic dissection. Eur Heart J.

[5] Hiratzka LF (2010). 2010 ACCF/AHA/AATS/ACR/ASA/SCA/SCAI/SIR/STS/SVM guidelines for the diagnosis and management of patients with thoracic aortic disease: a report of the American College of Cardiology Foundation/American Heart Association Task Force on Practice Guidelines, American Association for Thoracic Surgery, American College of Radiology, American Stroke Association, Society of Cardiovascular Anesthesiologists, Society for Cardiovascular Angiography and Interventions, Society of Interventional Radiology, Society of Thoracic Surgeons, and Society for Vascular Medicine. Circulation.

[6] Erbel R (2014). 2014 ESC Guidelines on the diagnosis and treatment of aortic diseases: document covering acute and chronic aortic diseases of the thoracic and abdominal aorta of the adult. The task force for the diagnosis and treatment of aortic diseases of the European Society of Cardiology (ESC). Eur Heart J.

[7] Chirillo F (2007). Clinical and anatomical characteristics of subtle-discrete dissection of the ascending aorta. Am J Cardiol.

[8] Chin AS (2018). Acute limited intimal tears of the thoracic aorta. J Am Coll Cardiol.

[9] Beller CJ (2004). Role of aortic root motion in the pathogenesis of aortic dissection. Circulation.

[10] Widder DJ (1983). Spontaneous nontraumatic rupture of the thoracic aorta. J Thorac Cardiovasc Surg.

[11] Padró JM (1988). Spontaneous rupture of the ascending aorta. J Cardiovasc Surg (Torino).

[12] Aoyagi S (1991). Spontaneous rupture of the ascending aorta. Eur J Cardiothorac Surg.

[13] Shkrum MJ, Silver MD (1992). Delayed rupture of spontaneous tear of the ascending aorta--report of two fatalities. Pathology.

[14] Handa N (1994). Spontaneous non-traumatic rupture of the thoracic aorta. Thorac Cardiovasc Surg.

[15] Comfort SR (1996). Sudden death while playing tennis due to a tear in ascending aorta (without dissection) and probable transient compression of the left main coronary artery. Am J Cardiol.

[16] Darbar D (2000). Localized aortic dissection: unusual features by transesophageal echocardiography. J Am Soc Echocardiogr.

[17] Akashi H (2003). Spontaneous rupture of the ascending aorta: case report and review. Circ J.

[18] Elefteriades JA (2005). Symptoms plus family history trump size in thoracic aortic aneurysm. Ann Thorac Surg.

[19] Vilacosta I (2009). Acute aortic syndrome: a new look at an old conundrum. Heart.

[20] Gomes de Farias LP (2021). Acute limited intimal tear of the right aortic sinus. Radiol Cardiothorac Imaging.

[21] Pomerance A (1977). The surgical pathology of thoracic aortic aneurysms. Histopathology.

[22] Roberts WC, Honig HS (1982). The spectrum of cardiovascular disease in the Marfan syndrome: a clinico-morphologic study of 18 necropsy patients and comparison to 151 previously reported necropsy patients. Am Heart J.

[23] Silver MD (1997). The healed and sealed aortic intimomedial tear. Cardiovasc Pathol.

[24] Roberts WC (2022). Aortic intimal-medial tear without dissection, the Marfan syndrome, and its forme fruste variety. Am J Cardiol.

[25] Morimoto N (2006). A localized intimomedial defect resulted in aortic regurgitation. J Thorac Cardiovasc Surg.

[26] Velasco CE (2018). Asymptomatic ascending aorta aneurysm with severe aortic regurgitation caused by multiple intimal-medial tears unassociated with aortic dissection. Am J Cardiol.

[27] Sarraj A (2017). How could these mini saccular aneurysms of ascending aorta be classified?. Ann Thorac Surg.

[28] Isselbacher EM (2022). 2022 ACC/AHA guideline for the diagnosis and management of aortic disease: a report of the American Heart Association/American College of Cardiology Joint Committee on Clinical Practice Guidelines. Circulation.

[29] Tsai TT (2006). Long-term survival in patients presenting with type A acute aortic dissection: insights from the International Registry of Acute Aortic Dissection (IRAD). Circulation.

[30] Azizzadeh A (2009). Blunt traumatic aortic injury: initial experience with endovascular repair. J Vasc Surg.

[31] Shalhub S (2014). Blunt abdominal aortic injury: a Western Trauma Association multicenter study. J Trauma Acute Care Surg.

[32] Shalhub S (2012). Blunt abdominal aortic injury. J Vasc Surg.

[33] Lin E, Alessio A (2009). What are the basic concepts of temporal, contrast, and spatial resolution in cardiac CT?. J Cardiovasc Comput Tomogr.

[34] Gavish L (2014). Inadequate reinforcement of transmedial disruptions at branch points subtends aortic aneurysm formation in apolipoprotein-E-deficient mice. Cardiovasc Pathol.

[35] Trachet B (2017). Angiotensin II infusion into ApoE^-/-^ mice: a model for aortic dissection rather than abdominal aortic aneurysm?. Cardiovasc Res.

[36] He C (2022). mTOR inhibition prevents angiotensin II-induced aortic rupture and pseudoaneurysm but promotes dissection in Apoe-deficient mice. JCI Insight.

[37] Rizzo S (2018). Sudden coronary death in the young: evidence of contractile phenotype of smooth muscle cells in the culprit atherosclerotic plaque. Int J Cardiol.

[38] Sundt TM (2007). Intramural hematoma and penetrating atherosclerotic ulcer of the aorta. Ann Thorac Surg.

[39] Nikolić S (2007). [Cardiac tamponade due to rupture of healed and sealed aortic intimomedial tear--case report]. Srp Arh Celok Lek.

[40] Komatsu S (2018). Angioscopic evaluation of spontaneously ruptured aortic plaques. J Am Coll Cardiol.

[41] Van der Wal AC (1993). Medial thinning and atherosclerosis--evidence for involvement of a local inflammatory effect. Atherosclerosis.

[42] Grewal N (2023). Are acute type A aortic dissections atherosclerotic?. Front Cardiovasc Med.

[43] Minelli S (2020). Reflections on atherosclerosis: lesson from the past and future research directions. J Multidiscip Healthc.

[44] Schwartz CJ (1988). Thrombosis and the development of atherosclerosis: Rokitansky revisited. Semin Thromb Hemost.

[45] Ross R, Glomset JA (1973). Atherosclerosis and the arterial smooth muscle cell: proliferation of smooth muscle is a key event in the genesis of the lesions of atherosclerosis. Science.

[46] López-Melgar B (2020). Short-term progression of multiterritorial subclinical atherosclerosis. J Am Coll Cardiol.

[47] Farb A (1999). Pathology of acute and chronic coronary stenting in humans. Circulation.

[48] Ross R (1993). Rous-Whipple award lecture. Atherosclerosis: a defense mechanism gone awry. Am J Pathol.

[49] Vilacosta I (2010). Acute aortic syndrome: a new look at an old conundrum. Postgrad Med J.

[50] Yousef S (2020). Diagnosis of thoracic aortic aneurysms by computed tomography without allometric scaling. JAMA Netw Open.

[51] Ziganshin BA (2015). Routine genetic testing for thoracic aortic aneurysm and dissection in a clinical setting. Ann Thorac Surg.

