# Supplementary material for: Intimomedial tears of the aorta heal by smooth muscle cell–mediated fibrosis without atherosclerosis
Source: JCI Insight. 2024 Apr 9;9(9):e172437. doi: 10.1172/jci.insight.172437 (PMC11141924; doi:10.1172/jci.insight.172437)
Supplement: Supplemental data [file jciinsight-9-172437-s061.pdf]

SUPPLEMENTAL MATERIALS

Intimomedial Tears of the Aorta Heal by Smooth Muscle Cell-Mediated Fibrosis without Atherosclerosis

Abdulrahman H.M. Hassab[1], David J. Hur[2, 3], Prashanth Vallabhajosyula[1], George Tellides[1, 3, 4, *], Roland Assi[1, 3, 4, *]

[1]Department of Surgery (Cardiac), Yale School of Medicine, New Haven, CT, USA.

[2]Department of Internal Medicine (Cardiovascular Medicine), Yale School of Medicine, New Haven, CT, USA.

[3]Veterans Affairs Connecticut Healthcare System, West Haven, CT, USA.

[4]Program in Vascular Biology and Therapeutics, Yale School of Medicine, New Haven, CT, USA.

*Denotes equal contribution.

Correspondence: Roland Assi, 330 Cedar Street, BB 225, New Haven, CT 06510, USA.

Phone: +1-203-785-5000; Fax: +1-203-785-3346; E-mail: roland.assi@yale.edu, or

George Tellides, 10 Amistad Street, 337B, New Haven, CT 06520, USA.

Phone: +1-203-737-2298; Fax: +1-203-737-6386; E-mail: george.tellides@yale.edu

Supplemental Materials:

Supplemental Table 1: Clinical features of individuals with intimomedial tears of the aorta

Supplemental Figure 1: Imaging of intimomedial tears

Supplemental Figure 2: Chronic, subacute, and acute tears of the aorta

Supplemental Figure 3: CD34 stem/progenitor cell marker in intimomedial tears

Supplemental Figure 4: Mitochondrial markers in intimomedial tears

Supplemental Figure 5: Collagen and elastic fibers in intimomedial tears

Supplemental Table 1: Clinical features of individuals with intimomedial tears of the aorta*.

**Demographics**<br>Age, Sex,<br>Ethnicity/Race	**Intimomedial Tears**<br>Number,<br>Maximal Size,<br>Location	**Aortic Disease**<br>Diameter (Asc),<br>Aneurysm/Ectasia,<br>Dissection,<br>Atherosclerosis	**Clinical History**<br>Aortic Symptoms;<br>Family History (Hx),<br>Genetic Diagnosis (Dx);<br>Comorbid Diseases	**CVD Risk Factors**<br>Hypertension (HTN),<br>Hyperlipidemia (HL),<br>Diabetes Mellitus (DM),<br>Smoker (current or ex)	**Aortic Valve**<br># of Leaflets;<br>Regurgitation<br>(AR),<br>Stenosis (AS)	**CT/TEE Imaging**<br>Specific Findings<br>(likely tear related);<br>Non-Specific Findings<br>(possibly unrelated)	**Surgery**<br>Aortic Procedures;<br>Associated Procedures
63 yr, male,<br>Non-Hispanic<br>Asian	2 tears, 20 mm,<br>anterior, proximal<br>ascending aorta	6.7 cm, aneurysm,<br>atherosclerosis	asymptomatic;<br>-family Hx, -genetic Dx;<br>atrial fibrillation	none	tricuspid;<br>moderate AR	CT: focal bulge,<br>TEE: systolic bulge	ascending/hemiarch replacement,<br>AVR; Cox-maze IV procedure
61 yr, male,<br>Non-Hispanic<br>White	2 tears, 5.5 mm,<br>non-coronary sinus,<br>mid aortic root	4.2 cm aortic root,<br>ectasia, 5.0 cm<br>Asc aorta, ectasia,<br>acute dissection	acute back pain;<br>-family Hx, -genetic Dx;<br>OSA	HTN, HL, DM<br>(all well controlled),<br>ex-smoker	tricuspid;<br>no AR/AS	CT: inadequate study,<br>TEE: no specific Abn;<br>bloody pericardial<br>effusions	root/ascending/hemiarch<br>replacement, AVR, coronary<br>artery reimplantation, TEVAR
32 yr, male,<br>Non-Hispanic<br>Black	3 tears, 18 mm,<br>anterior, proximal<br>ascending aorta	5.2 cm, aneurysm,<br>rapid aortic growth<br>(0.85 cm/yr x2 yr)	subacute chest pain &<br>HTN crisis 3 mo prior;<br>-family Hx, -genetic Dx;<br>Abd aorta dissection,<br>ESRD, cocaine use	HTN (uncontrolled),<br>ex-smoker	tricuspid;<br>severe AR	CT: inadequate study,<br>TEE: no specific Abn;<br>serous pleural &<br>pericardial effusions	root/ascending/hemiarch<br>replacement, AVR, coronary<br>artery reimplantation
77 yr, female,<br>Non-Hispanic<br>White	1 tear, 8.5 mm,<br>greater curvature,<br>proximal ascending<br>Aorta	5.6 cm, aneurysm	asymptomatic;<br>-family Hx, -genetic Dx;<br>Desc & Abd aortic<br>aneurysms, CAD	HL (well controlled),<br>ex-smoker	tricuspid;<br>moderate AR	CT: no specific Abn,<br>TEE: not visualized	ascending/hemiarch replacement,<br>AVR; CABG
61 yr, male,<br>Non-Hispanic<br>White	1 tear, 5.7 mm,<br>posterior, distal<br>ascending aorta	4.8 cm, ectasia,<br>acute dissection	chest and back pain;<br>-family Hx, -genetic Dx	HL (poorly controlled),<br>ex-smoker	tricuspid;<br>mild AR	CT: no specific Abn,<br>TEE: not visualized;<br>bloody pericardial<br>effusion	ascending/arch (zone 2)<br>replacement, aortic root repair,<br>AVR, TEVAR
57 yr, female,<br>Non-Hispanic<br>White	3 tears, 1 mm,<br>posterior, mid<br>ascending aorta	6.5 cm, aneurysm,<br>atherosclerosis	asymptomatic;<br>-family Hx, -genetic Dx;<br>Desc aortic aneurysm	HTN (well controlled),<br>ex-smoker	tricuspid;<br>moderate AR	CT: focal bulge,<br>TEE: not visualized	ascending/hemiarch replacement,<br>aortic valve repair
53 yr, male,<br>Non-Hispanic<br>White	1 tear, 4.4 mm,<br>non-coronary sinus,<br>upper aortic root	5.5 cm aortic root,<br>aneurysm, 4.5 cm<br>Asc aorta, ectasia	asymptomatic;<br>-family Hx, -genetic Dx;<br>CHF, OSA	HTN (well controlled),<br>DM (well controlled),<br>ex-smoker	tricuspid;<br>moderate AR	CT: no specific Abn,<br>TEE: systolic bulge;<br>serous periaortic fluid	root/ascending/hemiarch<br>replacement, AVR, coronary<br>artery reimplantation
40 yr, female,<br>Non-Hispanic<br>White	1 tear, 5.3 mm,<br>lesser curvature,<br>proximal ascending<br>aorta	5.6 cm aortic root,<br>aneurysm,<br>5.4 cm Asc aorta,<br>aneurysm	asymptomatic;<br>father: Asc aortic aneurysm,<br>patient: *COL5A1*, *SMAD6*,<br>and *HCN4* VUS	none	tricuspid;<br>moderate AR	CT: no specific Abn,<br>TEE: no specific Abn	root/ascending/hemiarch<br>replacement, AVR, coronary<br>artery reimplantation
70 yr, male,<br>Non-Hispanic<br>White	1 tear, 1.6 mm,<br>posterior, proximal<br>ascending aorta	5.3 cm, ectasia,<br>rapid aortic growth<br>(1.0 cm/yr x 1 yr),<br>PAU, atherosclerosis	asymptomatic;<br>-family Hx, -genetic Dx;<br>pectus excavatum	HTN (well controlled),<br>ex-smoker	bicuspid;<br>no AR/AS	CT: no specific Abn,<br>TEE: no specific Abn	root/ascending/arch (zone 1)<br>replacement, AVR, coronary<br>artery reimplantation

*Intimomedial tears were identified in 9 patients undergoing surgery with ascending aorta resection and are listed in chronological order of study inclusion. Subject characteristics and features of aortic disease were obtained from the electronic chart and intraoperative observations. Aortic root size is included when relevant for disease of that segment. Effusions diagnosed by imaging studies were confirmed as serous or bloody at surgery. Asc: ascending, Desc: descending, Abd: abdominal, PAU: penetrating atherosclerotic ulcer, -: negative, OSA: obstructive sleep apnea, ESRD: end-stage renal disease, CAD: coronary artery disease, CHF: congestive heart failure, VUS: variant of uncertain significance, CVD: cardiovascular disease, Abn: abnormality, AVR: aortic valve replacement, TEVAR: thoracic endovascular aortic repair, CABG, coronary artery bypass grafting.

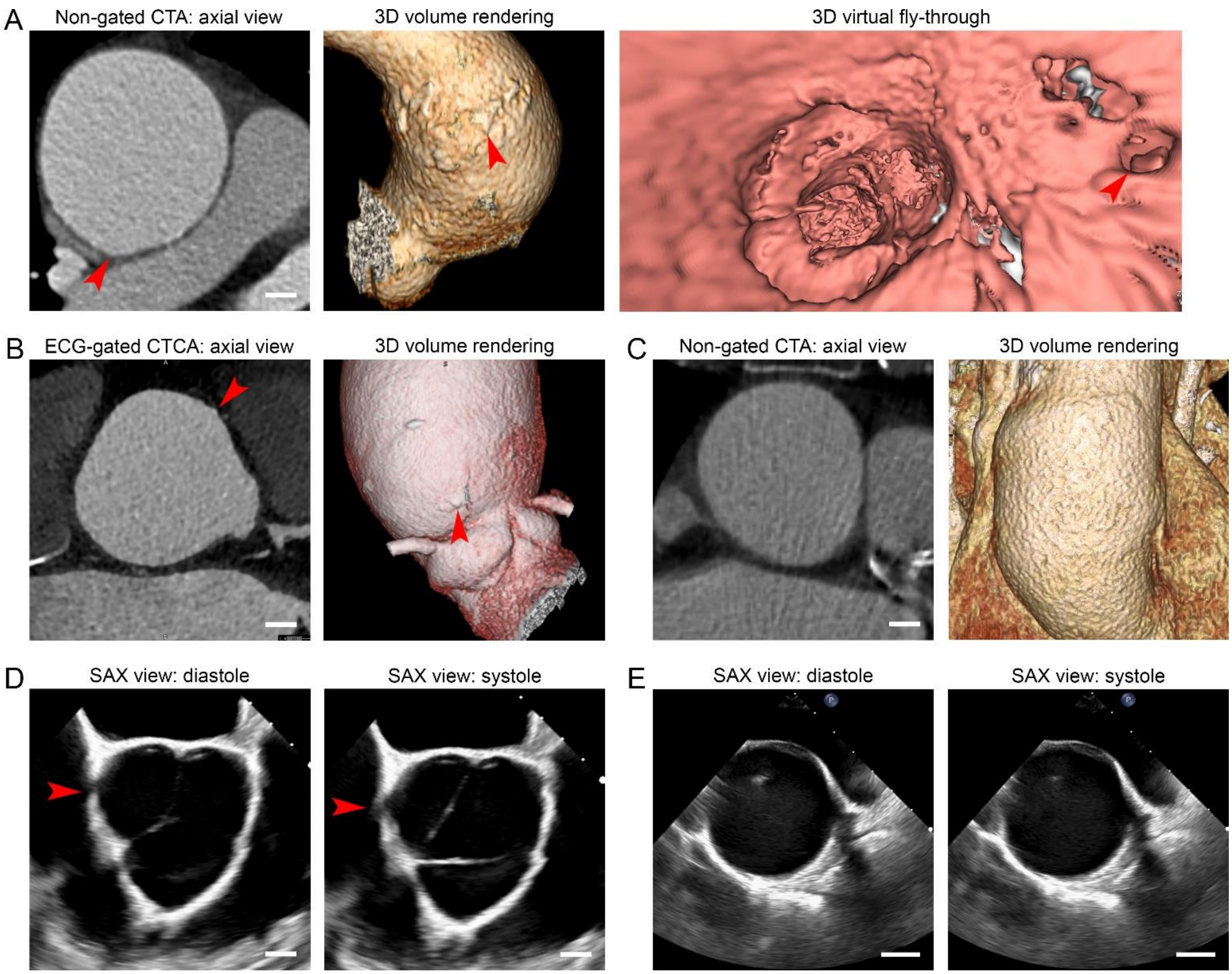

Supplemental Figure 1: Imaging of intimomedial tears. Subjects with intimomedial tears, identified by gross inspection and confirmed by histology, underwent preoperative CT scan and intraoperative TEE in preparation for thoracic aortic surgery. Routine clinical interpretation correctly diagnosed aneurysms and dissection but did not refer to subtle mural abnormalities corresponding to intimomedial tears that the investigators retrospectively identified in 3 of 9 patients (2 of 9 CT scans and 2 of 9 TEE). (**A**) Focal bulge (arrows) in posterior, mid-ascending aorta corresponding to site of intimomedial tear. (**B**) Focal bulge (arrows) in anterior, proximal ascending aorta corresponding to site of intimomedial tear. (**C**) Absence of mural abnormalities despite intimomedial tear in greater curvature of proximal ascending aorta. (**D**) Systolic bulge in noncoronary sinus of upper aortic root corresponding to site of intimomedial tear. (**E**) Absence of mural abnormalities despite intimomedial tear in posterior, proximal ascending aorta. Images represent axial views, 3D volume rendering, and 3D virtual fly-through of CT scans (non-gated or ECG-gated with intravenous contrast) or short axis views by TEE at end-diastole and end-systole. CTA: CT angiography, 3D: three-dimensional, ECG: electrocardiogram, CTCA: CT coronary angiogram, SAX: short axis. Scale bars: 1 cm.

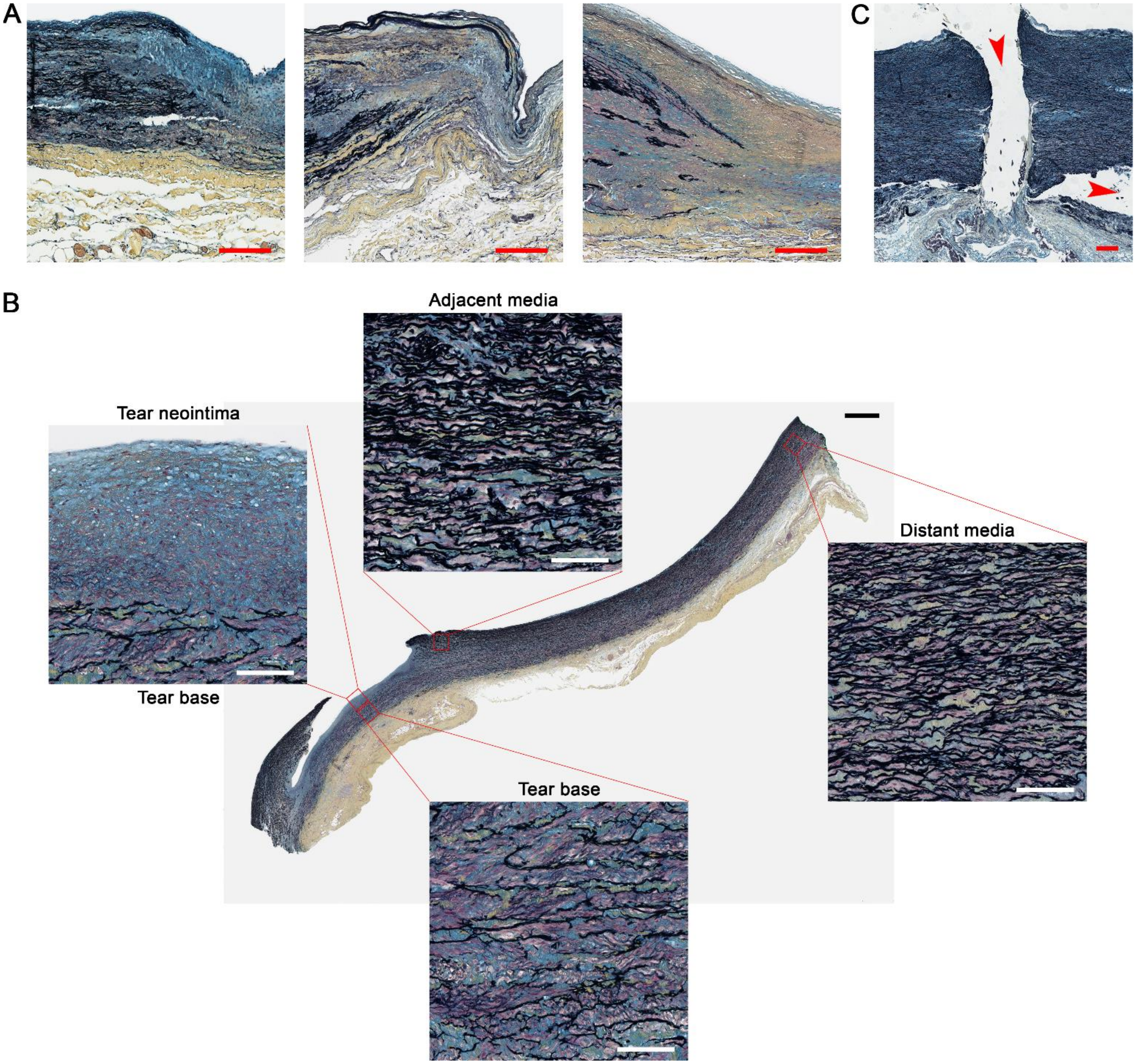

Supplemental Figure 2: Chronic, subacute, and acute tears of the aorta. Aortas with intimomedial tears or dissection were analyzed by histology using Movat's pentachrome stain. (**A**) Chronic, healed intimomedial tears from 3 specimens with varying fragmentation of elastic laminae (black color) in the adjacent media (left side) and absent elastic laminae but increased gylycosaminoglycans (blue color) or collagen (yellow color) in the tear neointima (right side). (**B**) Subacute, partially healed intimomedial tear with thin neointima covering the exposed media and free (non-adherent) edges formed by minimal (~3 mm) dissection of media on one side and no dissection of the other side. (**C**) Acute entry tear of dissection with narrow radial tear through the media (upper arrow) without tear neointima formation leading to substantial circumferential separation of the outer media (lower arrow). Orientation: internal (luminal) aspect above, external (adventitial) aspect below. Black scale bar: 1 mm, red scale bars: 200 μm, and white scale bars 100 μm.

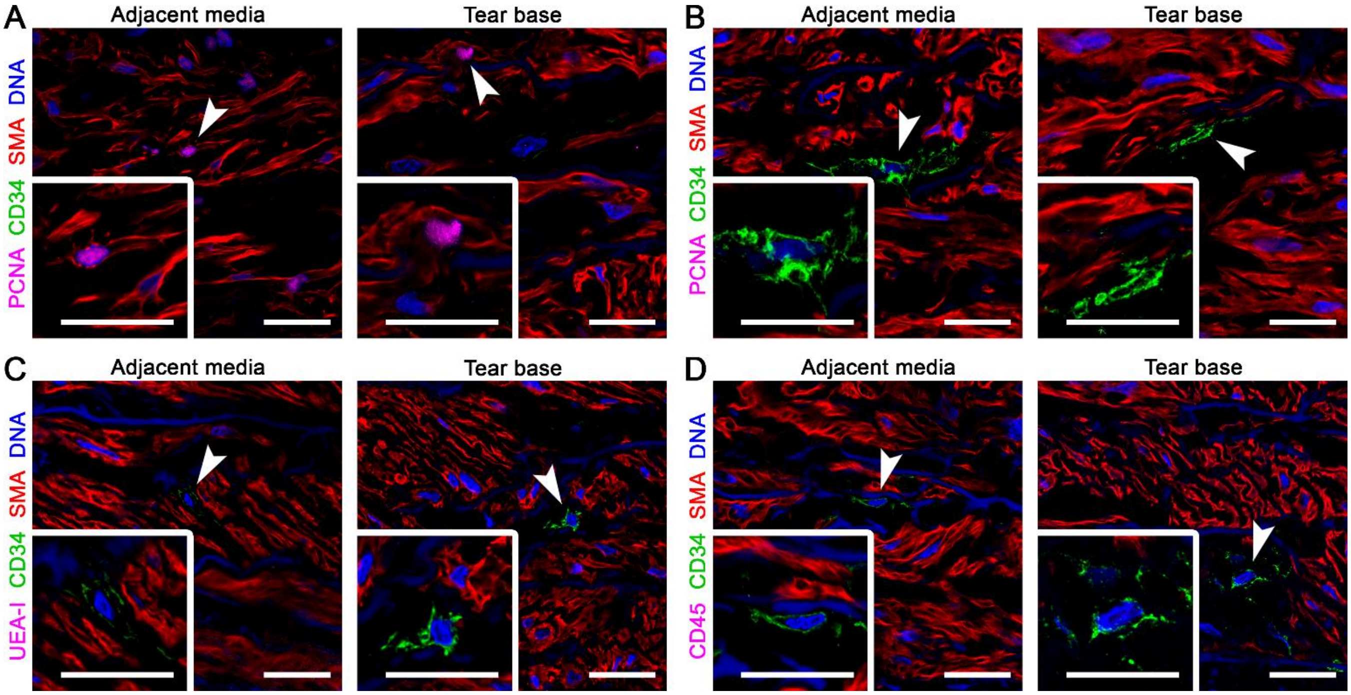

Supplemental Figure 3: CD34 stem/progenitor cell marker in intimomedial tears. The adjacent media and tear base of intimomedial tears containing occasional dividing cells were analyzed by immunofluorescence confocal microscopy with smooth muscle cells identified by SMA (red color) and nucleic DNA labelled with DAPI (blue color). (**A**) CD34 (green color) is not detected in dividing PCNA$^+$ (purple color) smooth muscle cells. (**B**) Rare CD34$^+$ medial cells are neither dividing (PCNA$^-$) nor smooth muscle cells (SMA$^-$). (**C**) CD34$^+$ cells are not labelled with the endothelial cell marker, *Ulex europaeus* agglutinin I (UEA-I, purple color). (**D**) CD34$^+$ cells are also not labelled with the leukocyte marker, CD45 (purple color). Insets display cells of interest (arrows) at higher magnification. Main panel scale bars: 25 μm; inset scale bars: 10 μm.

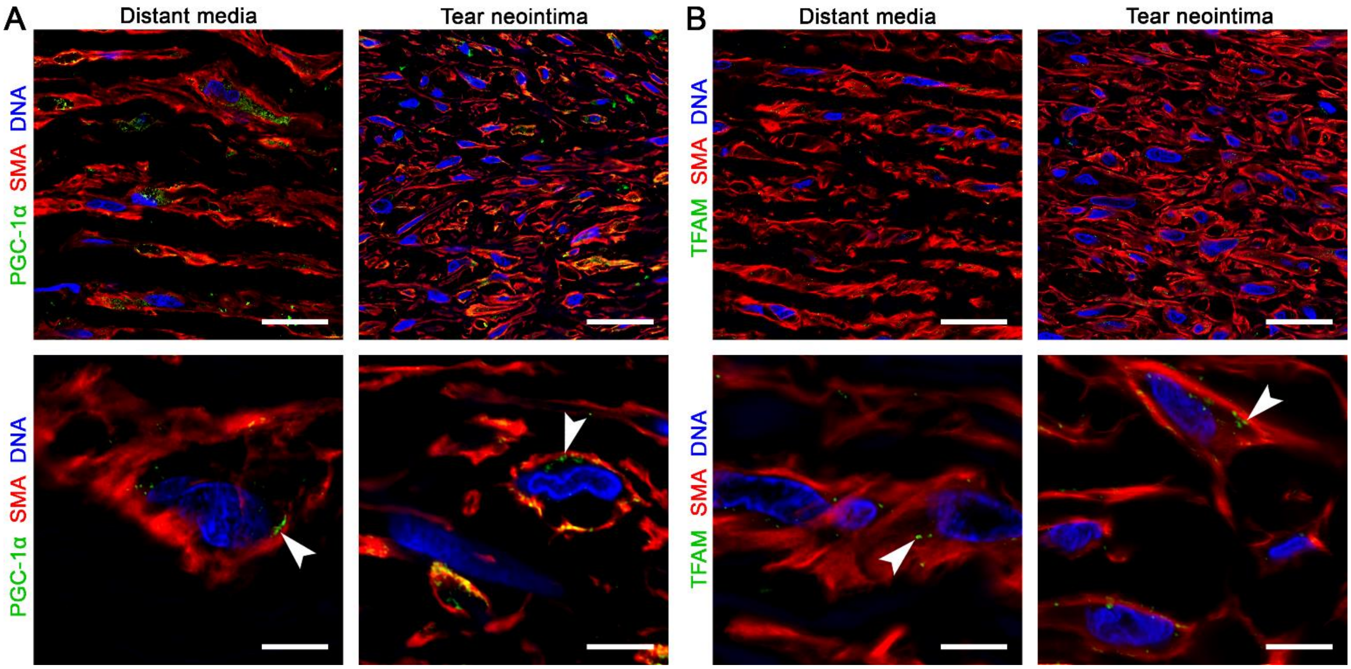

Supplemental Figure 4: Mitochondrial markers in intimomedial tears. The distant media and tear neointima of intimomedial tears were analyzed by immunofluorescence confocal microscopy with smooth muscle cells identified by SMA (red color) and nucleic DNA labelled with DAPI (blue color). Similar perinuclear punctate expression of (**A**) PGC-1α (green color), a regulator of mitochondrial biogenesis, and (**B**) TFAM (green color), a regulator of mitochondrial transcription, in both spindle-shaped smooth muscle cells of the distant media and smaller, irregular smooth muscle cells of the tear neointima. Bottom row displays mitochondria (arrows) of single smooth muscle cells at higher magnification. Upper row scale bars: 25 μm (1,000x magnification); bottom row scale bars: 5 μm (5,000x magnification).

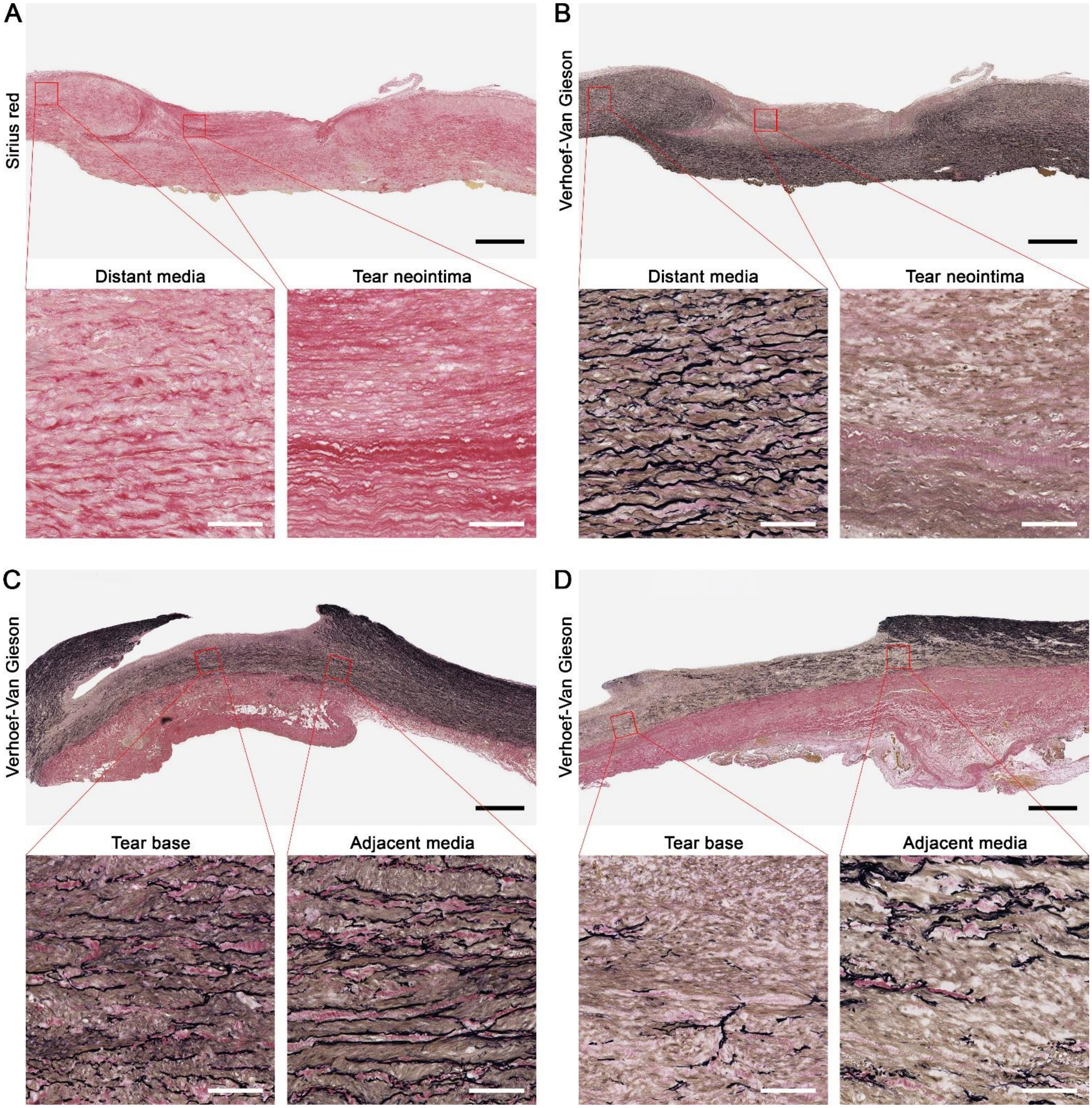

Supplemental Figure 5: Collagen and elastic fibers in intimomedial tears. Aortas with intimomedial tears were analyzed by histology. (**A**) Sirius red stain labeling collagen (red color) with higher magnification of selected areas. (**B**) Verhoef-Van Gieson stain labeling elastin (black color) with higher magnification of selected areas. Additional Verhoef-Van Gieson stains of two intimomedial tears from one aortic specimen in 32-year-old patient with (**C**) narrow or (**D**) wide defect showing greater fragmentation of outer elastic laminae in the tear base and adjacent media of the latter consistent with stretch injury. Orientation: internal (luminal) aspect above, external (adventitial) aspect below. Black scale bars: 1 mm; white scale bars: 100 µm.
